# Estimating the contribution of the porcine fecal core microbiota to metabolite production via mathematical modeling and *in vitro* fermentation

**DOI:** 10.1128/msystems.00366-23

**Published:** 2023-12-07

**Authors:** Salvatore Galgano, Helen Kettle, Andrew Free, Jos G. M. Houdijk

**Affiliations:** 1 Monogastric Science Research Centre, Scotland's Rural College, Edinburgh, Scotland, United Kingdom; 2 Biomathematics and Statistics Scotland, Edinburgh, Scotland, United Kingdom; 3 School of Biological Sciences, University of Edinburgh, Edinburgh, United Kingdom; University of Massachusetts Medical School, Brookline, Massachusetts, USA

**Keywords:** core microbiota, *in vitro* fermentation, mathematical modeling, microbiota, pig

## Abstract

The swine gut microbiota is a complex ecosystem found throughout the gastrointestinal tract, with multiple exchanges with the host and whose composition is linked to both external and internal factors, such as diet or breed. Diet, probiotic, or prebiotic interventions have been designed to boost beneficial host-microbiota interactions, such as the production of anti-inflammatory molecules, or the fermentation of otherwise undigested resources. In parallel, a smaller microbial population, shared among the same host species, independent of external or internal factors, has been described and defined as the “core microbiota.” Therapies targeting the core microbiota could possibly lead to more precise and long-lasting effects. However, the metabolic role of the porcine core microbiota, especially in relation to the rest of the microbial community, is currently missing. We present here the first dynamic model of the porcine core microbiota, which we used to estimate the core-microbiota metabolite production and to forecast the effect of a synbiotic intervention targeting the core genera of the core microbiota. We developed a community model in which a total of 17 microbial groups were established based on culture-based information of representative species. First, the model parameters were estimated, and the resulting model simulations were compared favorably with *in vitro* experimentation. The model was then used to predict the microbial dynamics of the core and non-core members under different experimental conditions. Therefore, it was able to theorize the main-metabolite core microbiota contribution, hypothesizing that it could be mainly responsible for acetate and propionate, but not for butyrate production.

**IMPORTANCE:**

Currently, little information is present in the literature to describe the generic metabolic role of the porcine core microbiota or to inform on the effect of interventions targeting the core genera. Moreover, both *in vitro* and *in vivo* experimentations aiming to explore the core microbiota dynamics are technically demanding, expensive, or restricted by ethical considerations. Modeling approaches can be used as an initial exploratory tool to develop hypotheses for targeted experimentation. Our mathematical model provides initial information on the microbial and metabolite dynamics of the core microbiota in relation to diet and therapeutic intervention.

## INTRODUCTION

Pork meat is highly produced (1), accounting for half of the European supply (2), and targeted swine gut microbiota manipulations are part of the currently applied strategies to improve health and performance (3). The porcine gastrointestinal microbiota, defined as the collection of microorganisms occurring in this anatomical environment (4), has multilevel interactions with the host. These include the provision of up to 30% of the energy requirements of a growing pig via metabolization to short-chain fatty acids (SCFAs) of host-undigested molecules (5) post-weaning dysbiosis leading to diarrhea and effects on feed efficiency (6) or fat metabolism (7). Prokaryotes are the main components of the pig gut microbiota (8); however, fungi, participating in the degradation of insoluble polysaccharides (9) and viruses, likely contributing to bacterial composition modulation (10), are also an integral part of this microbial ecosystem. Gut health and pork production are therefore directly related, underlying the importance of the pig gut microbiota in maintaining the host’s physical well-being (11).

Multiple intervention types have been designed (12) to modulate the microbial composition *in vivo*, e.g., the use of prebiotic, probiotic and fecal microbiota transplantation (13–15). However, long-lasting interventions on the pig microbiota can be hindered by the compositional variations reported between different host breeds (16, 17), age (18), and due to external factors, such as diet and hygiene level (19, 20). In an attempt to identify a more stable community among animals, a core microbiota (CM) has been described by different authors, in different animal species, whose composition seems to remain stable and predictable, notwithstanding different external and internal factors (21–25), which could facilitate the development of CM-targeted approaches (26). In parallel, a functional CM has also been proposed, identifying a series of functional microbial genes recurrently observed in the same group of hosts (27). The significance of the CM as a therapeutic target can be explained by the interactions between the CM and both the rest of the microbiota and the host, as a result of their coevolution (28). The main metabolic bacterial end-products are SCFAs, mainly the volatile acetate, propionate, and butyrate, but also the non-volatile formate, lactate, and some gases such as CO_2_, H_2_, and CH_4_ (29). Microbial compositional changes together with substrate availability, and physiological factors (e.g., pH), can determine both the concentration and type of microbial metabolites in the gut (30). The importance of the SCFAs, both physiologically and as a consequence of any intervention, is due to their interactions with the host; for example, acetate contributes to muscle energy reserves, propionate can readily be converted to glucose (31), and butyrate is the preferred fuel for colonocytes and has anti-inflammatory effects (32, 33).

A recent meta-analysis study aimed to investigate the possible presence of a CM in pigs, defining as core members the ones found in at least 90% of the analyzed samples (34); however, the CM-SCFA contribution within the whole microbiota has not yet been explored. As defined by Holman et al. (34), a CM could not exist in pigs according to the above definition; therefore, we developed the first dynamic mathematical model describing the growth-dynamics and main-SCFA production of the most abundant fecal genera, as per meta-analysis performed by (34, henceforth denoted H-CM), and based on a previous model simulating the dynamics of the human colonic microbiota (35, 36). Therefore, the CM definition used by us refers to the 16 genera with the highest relative abundance present in the fecal sample used in this study, also found within the genera with the highest relative abundance as informed by the meta-analysis by Holman et al. (34) as it can be found in Fig. 2B of their manuscript.

An *in vitro* continuous experiment was used to parameterize our porcine CM model, which predicted, *in silico*, the concentration over time of both bacteria and SCFAs when the former were growing under simulated nearly *in vivo* conditions. We used the model both to theorize the CM-SCFA contribution in the porcine lower intestine and to simulate a CM-targeting synbiotic intervention, therefore predicting the resulting CM-SCFA metabolism, which was characterized by increased acetate, propionate, and butyrate production, compared to our base-line comparison characterized by the pattern observed during an *in vitro* fermentation experiment. Our model, developed in the computer language R, is composed of 17 microbial groups (i.e., 16 CM genera and 1 group called “others” modeling the rest of the microbiota). The model is versatile and can be used as a tool to forecast the dynamics of porcine CM genera under different conditions. Although it is not meant to be a substitute for *in vitro* or *in vivo* investigations, our mathematical model could assist in identifying targeted hypotheses to be tested under these conditions. Moreover, our model is knowledge based and therefore expandable with the ever-advancing understanding of the relevant metabolic pathways included.

## RESULTS

### Compositional comparison between the literature meta-analysis CM and the current *in vitro* and *in silico* findings

We conducted a 35-day continuous fermentation experiment in which a swine fecal inoculum was grown under swine physiological conditions. We therefore compared the CM composition found in the three replicates of our continuous fermentation experiment (i.e., bioreactors) at time 0, with both the pooled fecal-sample inoculum and the fecal H-CM composition (Table 1). The microbial composition of the fecal inoculum was expected to somewhat vary from the composition observed in the fecal sample due to some of the genera not being able to thrive under the experimental conditions characteristics of our *in vitro* setup. When comparing the CM composition and relative abundance between the inoculum, the meta-analysis, and the bioreactor triplicates, the fecal CM accounted for 48.19% of the whole microbiota, which was similar to the 51% of the H-CM and to the 49.78% ± 4.5% (mean ± SD) of the triplicates. *Prevotella* was the most abundant genus throughout, with constant composition both *in vitro* and in the H-CM (~22%), whereas the next most abundant genera in the inoculum were *Megasphaera* (6.6%), *Streptococcus* (5.0%), RC9 (3.8%), *Clostridium* (3.4%), *Alloprevotella* (2.3%), and *Lactobacillus* (2.0%). However, less represented in the H-CM were *Treponema* (4.3%), *Succinivibrio* (3.3%), *Clostridium* (3.1%), *Lactobacillus* (2.9%), RC9 (2.4%), and *Blautia* (2.2%). The most noticeable differences between the inoculum and the bioreactors were recorded for *Megasphaera*, which were more abundant in the reactors (14% ± 4.6% vs 6.64%), with greater abundance in the first replicate, and the decreased abundance of both *Streptococcus* (2% ± 0.13%) and *Clostridium* (0.85% ± 0.08%) in the replicates compared to the inoculum (5.08% and 3.44%, respectively). The remaining genera were similarly represented in H-CM both inoculum and bioreactors, except for *Pseudobutyrivibrio*, *Bacteroides*, and *Sarcina*, which were not found in any of the samples analyzed in this study. On the other hand, we found that *Turicibacter* was somewhat present (~0.05%) in the inoculum and in the bioreactors; however, it was not described as a member of the fecal H-CM while the same meta-analysis located *Turicibacter* as a core genus in other gut locations.

**TABLE 1:** Core genera relative abundance in the inoculum, the three bioreactors at T0 compared to the H-CM (34) and the relative modeled genera

	This study					Reference 34
	Fecal inoculum	Reactor 1	Reactor 2	Reactor 3	*In-silico* CM	Fecal H-CM
*Prevotella*	22.27	22.45	29.48	19.16	23.58	22.86
*Megasphaera*	6.64	20.07	9.11	12.75	13.20	0.88
RC9	3.82	2.60	2.81	3.24	2.93	2.35
*Streptococcus*	5.08	1.33	1.41	2.10	1.68	0.78
*Lactobacillus*	1.95	2.40	1.17	1.91	1.76	2.93
*Alloprevotella*	2.33	1.94	2.06	1.73	1.90	1.76
*Clostridium*	3.44	0.78	0.82	0.96	0.87	3.13
*Treponema*	0.61	0.57	0.75	0.80	0.73	4.30
*Faecalibacterium*	0.62	1.01	0.96	1.39	1.15	1.17
*Succinivibrio*	0.05	0.05	0.03	0.10	0.07	3.32
*Blautia*	0.33	0.20	0.27	0.25	0.24	2.15
*Phascolarctobacterium*	0.29	0.61	0.50	0.50	0.53	0.98
*Ruminococcus*	0.63	0.24	0.29	0.28	0.27	1.17
*Parabacteroides*	0.04	0.03	0.04	0.03	0.03	0.98
*Pseudobutyrivibrio*	0.00	0.00	0.00	0.00	Not modeled	0.78
*Bacteroides*	0.00	0.00	0.00	0.00	Not modeled	0.78
*Escherichia*	0.01	0.00	0.02	0.01	0.01	0.39
*Sarcina*	0.00	0.00	0.00	0.00	Not modeled	0.29
*Turicibacter*	0.07	0.03	0.02	0.07	0.04	0.00
Others	51.81	45.70	50.25	54.71	51.00	49.00

### Model comparison with *in vitro* observations

**Fig 1:**
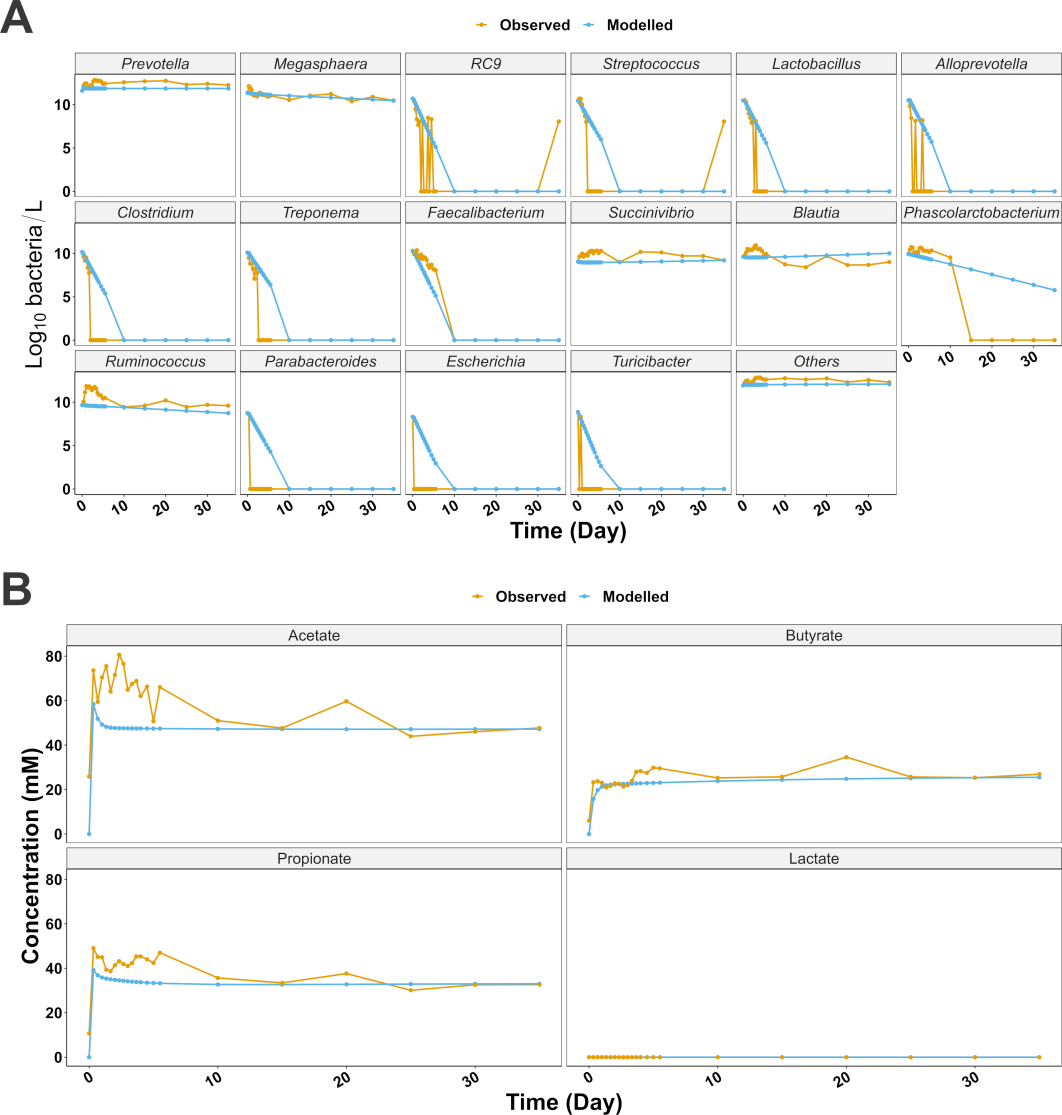
(A) Comparison between the predicted outputs of the CM model and the observations from the bioreactor experiment. The observed bacterial dynamics depicted here are the average of the observations among the three reactors. (B) Comparison between the predicted main SCFA pattern output of the CM model and the observations from the bioreactor experiment. The observed SCFA dynamics depicted here are the average of the observations among the three reactors.

As a first modeling step, we simulated the dynamics of the CM and of a group named “others” (i.e., rest of the microbiota), in conditions mimicking the bioreactors, allowing the comparison of the observed and modeled microbial and main SCFA concentrations (Fig. 1A and B). Some initial fluctuations characterized the observations of both bacteria and metabolites *in vitro*, as opposed to the modeled dynamics, which were more stable after the first hours of the simulated experiment. Both modeled and observed microbial/SCFA dynamics tended toward a steady state from day 10 of the experiment. As depicted in Table 2, the model showed a variable level of accuracy in forecasting the bacterial concentrations over time, typical of each comparison. Indeed, the *P* values for the linear regression (LM) between observations and predictions were <0.05 for all the genera apart from *Parabacteroides* (*P* = 0.09), *Escherichia* (*P* = 0.18), and “others” (*P* = 0.41). Nevertheless, the root mean square error (RMSE) was <1 log_10_ bacteria/L for 4 genera and for “others,” RMSE ≤5 log_10_ bacteria/L for 10 genera, and RMSE ≤10 log_10_ bacteria/L for 2 genera. Table 2 also summarizes the RMSE as a percentage of the mean observations (RMSE_%_), as a relative indicator of model performance, showing that a total of eight modeled groups achieved values <100%, indicating a high level of accuracy. Apart from “others,” only five core genera were found to be able to maintain a constant growth rate through the bioreactor experiment, e.g., *Prevotella*, *Megasphaera*, *Succinivibrio*, *Blautia*, and *Ruminococcus. In vitro*, *Prevotella* remained the most abundant genus with a concentration averaging 12.42 log_10_ bacteria/L after a small peak in the first 5 days, while the modeled predicted a more constant trend with an average of 11.85 log_10_ bacteria/L throughout (RMSE = 0.64 log_10_ bacteria/L). *Megasphaera* had a similar trend, both *in vitro* and *in silico* with observed and modeled average concentration being 11.13 log_10_ bacteria/L and 11.08 log_10_ bacteria/L, respectively (RMSE = 0.31 log_10_ bacteria/L). *Succinivibrio*, *Blautia*, and *Ruminococcus* showed higher concentration variations throughout the *in vitro* fermentation, with average values of 9.86 log_10_ bacteria/L, 9.91 log_10_ bacteria/L, and 10.72 log_10_ bacteria/L. The model was able to depict the average concentration of 9.00 log_10_ bacteria/L for *Succinivibrio* (RMSE = 0.96 log_10_ bacteria/L), 9.62 log_10_ bacteria/L for *Blautia* (RMSE = 0.90 log_10_ bacteria/L), and 9.44 log_10_ bacteria/L for *Ruminococcus* (RMSE = 1.47 log_10_ bacteria/L). While the model depicted well the dynamics of the group “others” with RMSE of 0.54 log_10_ bacteria/L, the accuracy of this prediction was lower than that of the rest of the genera according to the LM (*P* = 0.41). This group recorded a constant concentration throughout both observations and modeled values, with average log_10_ concentrations of 12.50 log_10_ bacteria/L and 12.02 log_10_ bacteria/L, respectively. On the other hand, *Phascolarctobacterium* concentration was found to be 0 log_10_ bacteria/L at day 35 of the *in vitro* experimentation, while, although somewhat decreasing toward day 35 in the model, its final concentration was 5.77 log_10_ bacteria/L, indicating deviating modeled dynamics compared to the observations (RMSE = 3.41 log_10_ bacteria/L).

**TABLE 2:** RMSE calculated between the observations and the predictions of both genera and main SCFAs throughout the 35 days

Genus compared	RMSE (log_10_ bacteria/L)	RMSE_%_ (% of observation means)	LM (*P* value)	SCFA compared	RMSE (mM)
*Prevotella*	0.64	5.13	0.02	Acetate	18.08
*Megasphaera*	0.31	2.78	<0.01	Butyrate	4.18
RC9	4.44	111.68	<0.01	Propionate	7.90
*Streptococcus*	5.07	146.97	0.02	Lactate	0
*Lactobacillus*	3.92	104.78	<0.01		RMSE_%_ (% of observation means)
*Alloprevotella*	5.63	274.90	0.02	Acetate	29.68
*Clostridium*	4.78	192.97	<0.01	Butyrate	17.11
*Treponema*	4.55	145.88	<0.01	Propionate	20.11
*Faecalibacterium*	1.43	21.04	<0.01	Lactate	
*Succinivibrio*	0.96	9.72	0.03		LM (*P* value)
*Blautia*	0.90	9.11	<0.01	Acetate	<0.01
*Phascolarctobacterium*	3.41	42.94	<0.01	Butyrate	<0.01
*Ruminococcus*	1.47	13.70	<0.01	Propionate	<0.01
*Parabacteroides*	5.25	666.21	0.09	Lactate	
*Escherichia*	4.89	1294.61	0.18		
*Turicibacter*	4.63	592.52	0.05		
Others	0.54	4.31	0.41		

The model performed rather well in predicting the steady-state main-SCFA concentration (Fig. 1B), with *P* value of the LM calculated between the predictions and the observations <0.01 for acetate, propionate, and butyrate. Time 0 modeled metabolite concentration was 0 mM; however, observed acetate, propionate, and butyrate concentrations were 25.88, 10.68, and 6.00 mM, respectively, while lactate remained undetected both *in vitro* and *in silico* (RMSE = 0 mM). As described above, the model did not predict fluctuations before reaching steady state; however, it was rather effective in predicting both the average and steady-state abundance. Acetate recorded the highest degree of variation until day 5.5, with concentration ranging from 50.77 mM to 80.62 mM before reaching a somewhat constant value of ~50 mM from day 10 to day 35 (47.69 mM). Modeled acetate, on the other hand, rapidly reached steady state after an initial peak at 58.4 mM (day 0.3), with constant levels at ~47.00 mM until day 35, when the final predicted concentration was 47.22 mM (RMSE = 18.08 mM). Propionate showed more fluctuations *in vitro* between 8 hours and day 5.5, with concentrations ranging between 38.72 mM (day 1.7) and 49.04 mM (day 0.3) before reaching a pseudo-steady state around day 10 (35.68 mM) with the final observed concentration (day 35) of 32.68 mM (RMSE = 7.90 mM). Observed butyrate reached ~21 mM from day 0 to day 3, before peaking at ~28 mM around day 4, with somewhat constant concentration toward day 35 (26.9 mM); on the other hand, the model predicted a single peak at ~21 mM around day 1, with concentration slowly increasing, reaching 25.5 mM at day 35 (RMSE = 4.18 mM).

### Evaluation of the CM metabolite contribution under simulated nearly *in vivo* conditions

**Fig 2:**
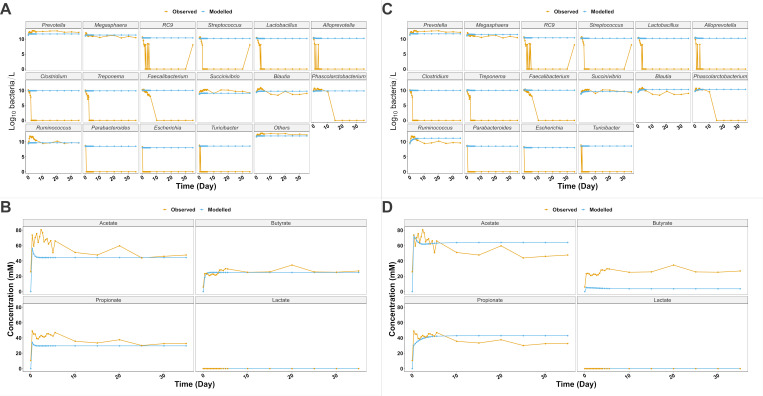
(A) Microbial dynamics when modeling “nearly *in vivo*-like” conditions, with the concentration of all the groups constant through the simulation and with higher strain trait variability with two strains per modeled group. (B) Main SCFA dynamics when modeling “nearly *in vivo*-like” conditions, with the concentration of all the groups constant through the simulation and with higher strain trait variability with two strains per modeled group. (C) Microbial dynamics of the core genera only simulating nearly *in vivo*-like concentration, without the contribution of the rest of the microbiota (others). (D) Main SCFA dynamics as product of the core genera only, when simulating nearly *in vivo*-like concentration, without the contribution of the rest of the microbiota (others).

To postulate the role of the CM within the gut microbiota and under porcine colon physiological conditions, we simulated an *in vivo*-like (nearly *in vivo* conditions) scenario, characterized by the presence at the end of the simulations of all the genera and with two strains for each of the 17 groups, but without taking into account of biotic factors, SCFA absorption, immune system contribution, and host-microbiota dynamics. All the simulations represented 35-day dynamics to be consistent with the set-up conditions as described in the previous section when comparing the model to *in vitro* data. Thus, three different conditions were modeled, (i) CM genera + “others” (ii), CM genera only, and (iii) “others” only. When modeling CM + “others” (Fig. 2A and B), the modeled concentration of all the microbial groups was constant from day 0 to steady state, lactate was not detected throughout the 35 days, and acetate, propionate, and butyrate steady-state concentration were 44.4, 29.7, and 24.9 mM, compared to the observed 47.7, 32.7, and 26.9 mM, respectively, with associated RMSE values of 20.51 (RMSE_%_ = 33.66%, LM *P* = 0.002), 11.64 (RMSE_%_ = 29.64%, LM *P* < 0.001), and 3.65 (RMSE_%_ = 14.93%, LM *P* < 0.001), respectively.

The model was then run with the CM genera only to estimate, *in silico*, their main metabolite contribution. Bacterial dynamics remained comparable to the above-described simulation, with constant concentrations throughout (Fig. 2C). In general, CM metabolite contribution was linked to greater proportions of acetate and propionate and smaller concentrations of butyrate compared to those observed (Fig. 2D). Acetate modeled dynamics were characterized by an initial peak at 73.85 mM at 8 hours, followed by a decrease in concentration toward ~62 mM at 1.67 days, with 64 mM at day 35. *In vitro*, apart from the initial increase in the concentration of 73.67 mM at 8 hours, observed steady-state concentration was 34% smaller, resulting in RMSE = 12.2 (RMSE_%_ = 20.02%, LM *P* = 0.005). We observed a similar scenario for propionate, whose modeled initial concentration at 8 hours was 30.38 mM, while steady-state concentration was 31.5% greater (43 mM) than the observed one of 32.7 mM (RMSE = 7.93 mM, RMSE_%_ = 20.20%, LM *P* = 0.005). On the other hand, smaller values were predicted for butyrate throughout the simulation, with concentration reducing from 5.34 mM at 8 hours to 3.68 mM at day 35, which was 86.3% less than the observed 26.9 mM at day 35 (RMSE = 20.85 mM, RMSE_%_ = 85.29%, LM *P* = 0.029).

**Fig 3:**
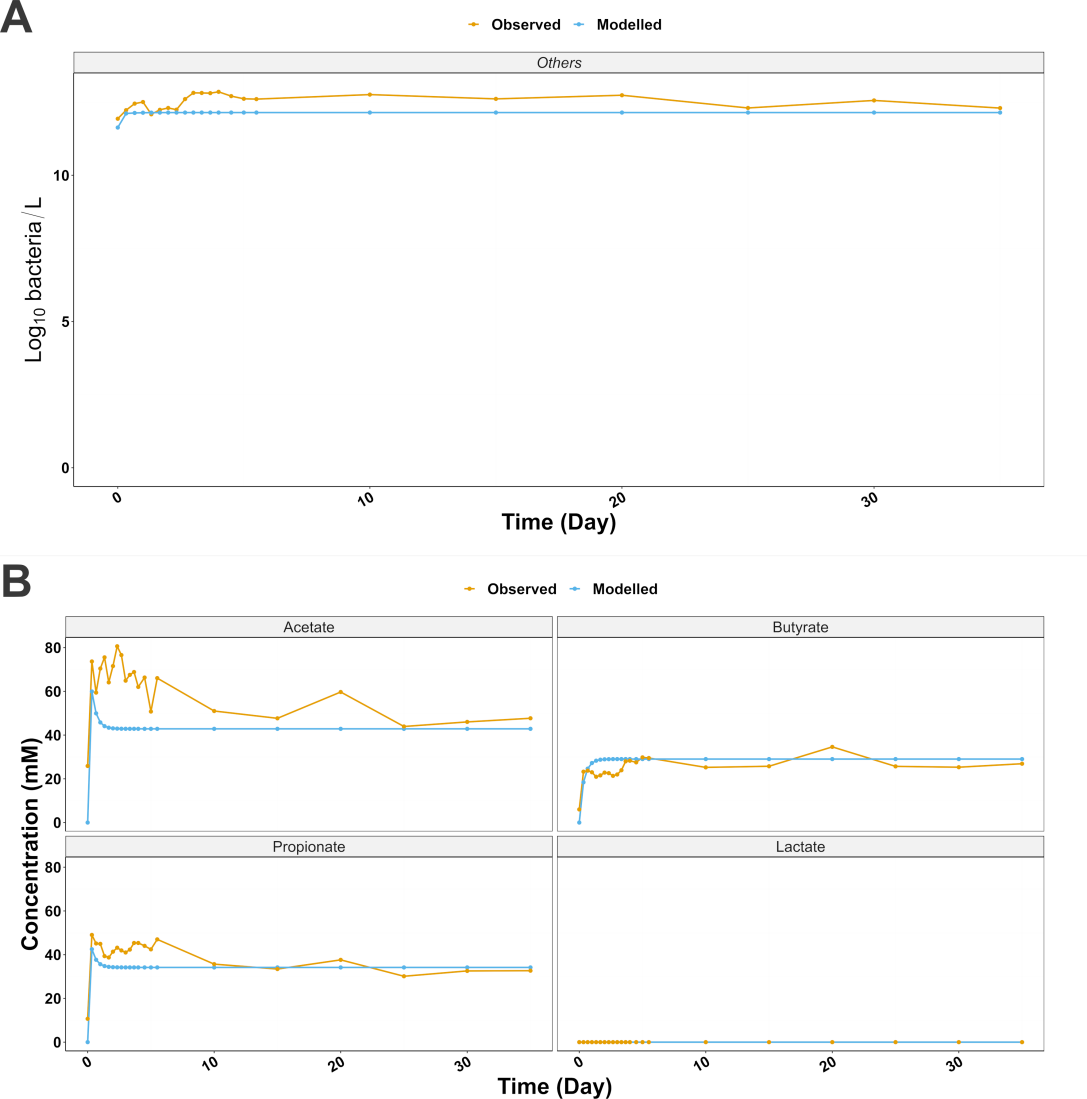
(A) Others modeled without the contribution of the core genera. (B) Main SCFA dynamics produced by others during a 35-day simulation mimicking the conditions found in the bioreactor.

Finally, the model was run with “others” only to simulate the theoretical contribution of the rest of the microbiota if CM was not present. The concentration of “others” during the simulation was rather analogous to the *in vitro* observations (Fig. 3A, RMSE = 0.45 log_10_ bacteria/L, RMSE_%_ = 3.56%, LM *P* = 0.017), with modeled and observed steady-state log_10_ bacteria/L concentrations of 12.2 and 12.3, respectively. The comparison of the main metabolites indicated a similar pattern between model and observations (Fig. 3B), with steady-state concentrations of acetate, propionate, and butyrate measuring 42.9 (RMSE = 21.36 mM, RMSE_%_ = 35.05%, LM *P* = 0.002), 34.2 mM (RMSE = 7.56 mM, RMSE_%_ = 19.25%, LM *P* < 0.001), and 29.0 mM (RMSE = 4.7 mM, RMSE_%_ = 19.24%, LM *P* < 0.001), respectively.

### *In silico* experimentation

**Fig 4:**
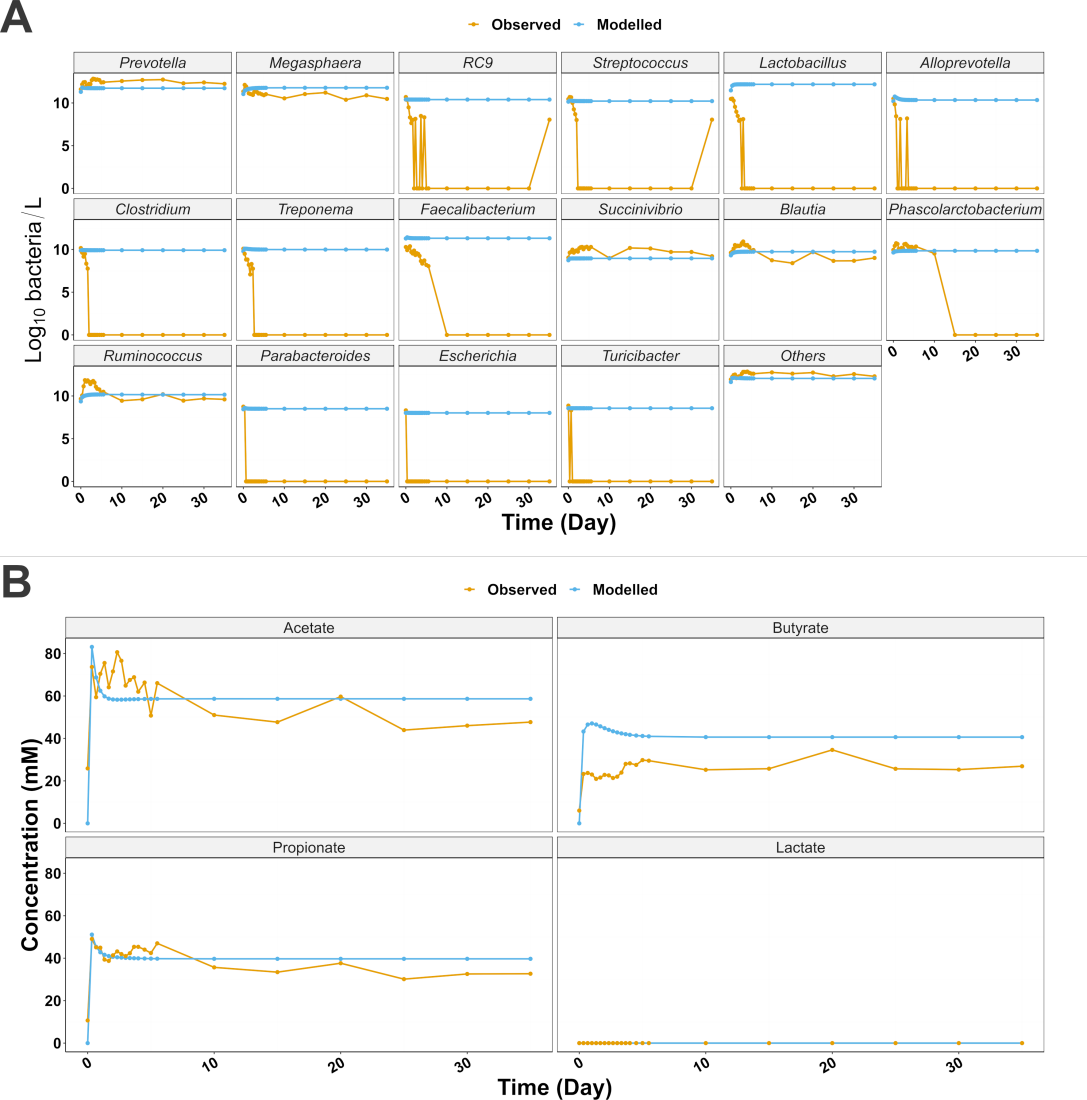
(A) Microbial dynamics of a simulation of a synbiotic intervention with *Lactobacillus*, *Faecalibacterium*, and resistant starch. (B) SCFA dynamics of a simulation of a synbiotic intervention with *Lactobacillus*, *Faecalibacterium*, and resistant starch.

Finally, we simulated a synbiotic continuous intervention with both microbial and substrate concentration enhancement of 20× *Lactobacillus* (2.73 g/L), 20× *Faecalibacterium* (1.78 g/L), and 5× resistance starch (21.6 g/L). The results are depicted in Fig. 4A and B, showing the increased concentration of both *Lactobacillus* and *Faecalibacterium* as a consequence of their probiotic-simulated use, while the rest of the microbial dynamics were similar to the previous simulations. On the other hand, the simulated intervention resulted in an increased concentration of acetate, propionate, and butyrate, while lactate remained undetected throughout. Modeled acetate (RMSE = 12.24 mM, RMSE_%_ = 20.09%, LM *P* = 0.003) had an initial peak at 8 hours of 83.11 mM, before tending to lower steady-state values of 58.66 mM, with a 23% increase compared to the observations. Propionate simulations also recorded a more modest increase with an 8-hour peak of 51.12 mM and a 21.4% higher day 35 concentration of 39.7 mM (RMSE = 4.94 mM, RMSE_%_ = 12.58%, LM *P* < 0.001). Similarly, modeled butyrate had an initial peak at 43.26 mM, with day 35 concentration of 40.6 mM, 51% higher than the observed values (RMSE = 17.7 mM, RMSE_%_ = 72.69%, LM *P* < 0.001).

## DISCUSSION

The swine GI tract CM is a promising target for long-lasting interventions, such as probiotics and prebiotics, due to the relatively constant abundance of the CM genera across the same host species (37). Although the microbiota is compositionally well defined, there is a lack of predictive tools that can inform on metabolite production, especially in response to external interventions (38). Therefore, aiming to predict the CM main-metabolite production, we developed the first dynamic model of the porcine fecal CM, based on the description of the composing genera by a meta-analysis study (34). It is important to mention that not all the fecal genera with the highest relative abundance as found by Holman et al. (34) through their meta-analysis, identified here as H-CM, were found in our pooled fecal sample. However, the composition of the latter was found to reflect the generic consensus as per literature studies (18, 39).

Due to its architecture, our model inevitably maintained a certain degree of uncertainty related to both some kinetic parameter values, based on theoretical considerations, and the stoichiometry, which in some cases could not depict the whole genus complexity by basing their data frame only on one or few representative species. However, our approach was consistent with community-level dynamic parameters derived from monocultures (40). Moreover, the closeness between the predictions and the observations of both the representative species (Tables S1–S20) and the bioreactors point toward a significant degree of reliability of the CM model outputs. It ought to be acknowledged, however, that a further limitation of the current model is represented by the selection of parameters from culture-based information of representative species. This modeling approach led to narrow the parameter selection toward those values likely to be observed in pure cultures, and therefore, it did not consider the competitive microbial dynamics or *in vivo* interactions, which could affect the general model dynamics. Nevertheless, it is currently rather challenging to gather information on single-strain dynamics of bacteria growing in a complex environment or *in vivo*, hence the use of culture-based data to inform the model.

The integration of our empirical data with our model provided a further level of validation when comparing the model outputs and when predicting the potential CM role, as this approach has been described to lead to more accurate predictions of microbial dynamics applicable to different simulated environments (41). Different strategies can be adopted when modeling different complex ecosystems, including a spatially explicit individual-based model of bacterial growth, able to anticipate substrate demand and competition on two dimensions (42), or a compartmentalized modeling approach to carbohydrate degradation by human colonic microbiota (43). The latter allowed the integration of intestinal physiology with microbial metabolism, while our CM model currently does not include host factors.

According to our findings, the CM main metabolite production could be characterized by producing more acetate (+34.0%) and propionate (+31.5%) and especially less butyrate (−86.3%) relative to that observed for the whole microbiota. Interestingly, the predicted metabolic role of “others” in the absence of a CM was similar to the observations, possibly indicating a marginal role of the CM in the total bacterial dynamics. However, it cannot be excluded that this could have been due to the model architecture of the group “others.” The latter was organized in functional groups representative of the whole microbiota based on the 10 described metabolic functional groups in a previous human colonic microbiota model (35). Moreover, although the approach captures most gut species, it should be noted that lipolytic or a-saccharolytic genera like *Anaerovibrio* (44), *Adlercreutzia* (45), or H_2_ depletion by sulfate-reducing bacteria (46) were not considered in these 10 groups.

Our model was effective in predicting the main-metabolite pattern observed in the bioreactors, with highly similar microbial dynamics and average steady-state metabolite production. Modeled and observed lactate concentration was constantly undetected, which was in accordance with *in vivo* and *in vitro* findings (47). Although the observed acetate, propionate, and butyrate ratios as approximately 45:30:25 differ from the often observed 60:25:15 under *in vivo* conditions (48), they did accord with those observed *in vitro* using varying substrates (49, 50). Likely our observed lower acetate levels could have been linked either to the pH range used instead of fixed values, with lower pH values responsible for higher butyrogenic tendencies (51) or to the substrate availability and consequent microbial dynamics in the bioreactors. It must be acknowledged that other possibly contributing metabolites forecasted by the model (e.g., formate) were not measured.

Some of the genera, especially belonging to the CM, did not prevail *in vitro* with their concentrations rapidly reducing below detection limits throughout the continuous fermentation. This was not unexpected due to the fastidious nature of some of the genera, indicating that some of the microorganisms present in the inoculum would have required different and *ad hoc* culture conditions (52), whose growth rate was lower than the dilution rate did not allow them to proliferate over time in the main vessel. *Lactobacillus spp.* is reported as almost ubiquitous *in vivo* (18); therefore, its observed decline in our *in vitro* system could be explained either with the competition with other microbiota members or with the fact that *in vivo Lactobacillus* is likely supplied from the stomach (53), whose contribution was not accounted for in our setup. It must also be acknowledged that we used a fecal inoculum to describe the fecal CM dynamics while comparing the modeled outputs to observations of an *in vitro* environment simulating colonic conditions. This was due to the lack of dynamic continuous conditions within the fecal environment, the accessibility toward the fecal sample type instead of the colonic ones, and based on the similarity assumptions of the microbiota in the two locations (54). However, the comparison between the modeled and predicted values was considered as a starting point to validate and to test the ability of the model to predict both microbial and metabolite dynamics in a determined environment. Therefore, the output of our study is the predicted metabolic role of the CM within the lower gut, based on validations through observations of the fecal CM growing under physiological colonic conditions. As a consequence of the validation process undertaken, our modeling tool could be used to simulate different environmental conditions to answer to different research needs.

Although the detailed mechanism of action is yet to be fully described, probiotic and prebiotic interventions in pigs affect the final microbial fermentation pattern toward positive health outcomes (3) and tend to reduce colonization by pathogens (55). Here, we simulated the effect of a synbiotic therapy (*Lactobacillus*, *Faecalibacterium*, and resistant starch combined) on the main metabolite pattern. When comparing the simulated acetate-propionate-butyrate production resulting from the synbiotic intervention to the observations of the same community, growing *in vitro*, not exposed to the synbiotic treatment, we showed a 23% greater acetate production, a 21.4% greater propionate production, and a 51% greater butyrate production due to the synbiotic intervention. The rationale behind this proposed intervention was to include a lactate producer via heterofermentative metabolism, which also produced acetate (56). The latter could be used, together with RS by *Faecalibacterium* to produce butyrate, normally considered a positive health marker (32). Our modeling approach could give the first indications on the microbial dynamics following the proposed intervention *in vivo*, and it would lay the foundations for further *in vitro*/*in vivo* experimentation.

In conclusion, our model not only suggested that the role of the CM *in vivo* could be tending toward acetate and propionate production, with a possibly secondary role in butyrate production, but CM genera could also represent an optimal therapeutical target due to their constant presence among the same host species. Our modeling tool could assist both *in vitro* and *in vivo* experimentation, allowing forecasting effects of different environmental conditions or interventions to the resulting fermentation pattern.

## MATERIALS AND METHODS

### Model design and mathematical formulation

The model was assembled and solved in microPop V1.6 (35) in R (57) and simulated a continuous fermentation experiment in which the microbial groups (i.e., CM-genera and “others”) were growing in an anaerobic bioreactor, connected to a flow-in and a flow-out, with known dilution rate, pH, and medium composition. Each microbial group was organized in data frames summarizing resource/metabolite stoichiometry and type, together with the values for the maximum growth rate (*µ*_max_), half-saturation constant (*K*), and yield (*Y*; Tables S1–S20).

The values for *µ*_max_ and *Y* were mostly based on the theoretical considerations as per previous versions of the model for the colonic human microbiota (35, 36) while taking into consideration culture-based data relative to a representative species for each genus, where these were available. In particular, while a full description of parameter estimation is provided in the original manuscripts (35, 36), theoretical energy gain calculations were used to estimate *Y* in such a way that, for example, it was estimated that hexose consumption would generate 2.8–4 ATP molecules and that, for example, a yield of 0.333 (*g ∙ g*^−1^) would be the result of sugars/starch fermentation.

The selection of the values for *K* was based on empirical parameter estimation using the observations collected during the continuous fermentation experiment carried out and described here. In particular, initially, the *K* value for each CM was set to 0.001 g/L as per original manuscripts and as per microPop package (35, 36); thus, the assembled CM model was run to simulate the experimental conditions of the bioreactor *in vitro* setup as described in the relevant sections of this manuscript (i.e., initial conditions of resistant starch = 4.32 g/L, non-starch polysaccharides = 7 g/L, proteins = 15 g/L, simple sugars = 2 g/L, acetate = 0 g/L, propionate = 0 g/L, butyrate = 0 g/L, lactate = 0 g/L, and dilution rate = 0.111 h^−1^). Thus, the model was run multiple times, and the *K* values for each group were changed according to biologically compatible values (58) until empirically obtaining growth dynamics and fermentation patterns that could be aligned to the observations of the *in vitro* experimentation on which the comparison was based. The final *K* values for each CM are summarized in Tables S1–S20, together with the rest of the parameters selected for each of the CM and “others.” It must be noted that the initial metabolite concentrations (e.g., acetate, propionate, and butyrate) were by default set to 0 g/L in the model as it was assumed that there was no metabolite production in the absence of bacterial growth. Although, as described in the results, we did record a non-zero concentration for acetate, propionate, and butyrate in the reactors, but since these could have been due to previous (i.e., *in vivo* metabolite production) fermentation events, we decided to set the initial modeled concentration to 0 g/L.

Four ascending pH values (”pH corners” in microPop) were included in each data frame, representing either the optimal pH, corresponding to maximum growth (i.e., the two middle values in pH corners), or the extreme pH, above and below growth would not be possible. The modeled starting concentration of both microbial groups and resources/metabolites and the values for the dilution rate and pH could be edited at the beginning of each simulation. In this way, the model was used to (i) simulate and compare the observed *in vitro* dynamics and carry out model parametrization, (ii) evaluate the main-SCFA core contribution while allowing for continuous flow-in of the microbial groups, and (iii) increase the concentration of selected resources or microbial groups simulating a synbiotic treatment. In microPop, the model was run via solving a system of ordinary differential equations (ODEs), based on the Monod equation for the growth of microorganisms, where the resources were classified into essential (Se), substitutable (S), water-resource (Sw), and boosting (Sb). An extensive description of the ODEs used in microPop can be found in the manuscript (35), whereas an example of a generic equation that summarizes the rate of production of one generic metabolite, as per the original “microPop” manuscript, can be found below. Therefore, according to the equation used in the R package, the production of the metabolite *z*, in a system in which there are *N_z_* metabolites and *N_i_* substrates, the production rate *M* (gl^−1^h^−1^) of *z* by the CM *j* is given by the following:


Eq. 1 $$\frac{m_{z}n_{z}}{\sum_{k=1}^{N_{z}}m_{k}n_{k}}\left(\sum_{i=1}^{Ni}U_{ji}+U_{jw}-\mu_{ji}\left(t\right)\right)\;,$$

where *m_k_* is the molecular mass and *n_k_* is the number of molecules of product *k*. In this way, the microbial growth rate (*µ_ij_*) is subtracted to conserve mass, also computing the uptake of water (*U_w_*).

Currently, the model comprises 34 state variables (i.e., 16 CM genera, the bacterial group called “others,” 4 main resource types, and 13 metabolites among which some can function also as resources) and 4 parameters (*µ*_max_, *K*, *Y*, and pH corners). A full list of the resources and the metabolites modeled and their organization in the 17 data frames can be found in Table S1.

The output of the model was the rate of change (i.e., g/L concentration per unit time) of the resources, microbial groups, and metabolites. The latter was converted to mM per unit of time for comparison with gas chromatography observations, via using the molar mass of the different metabolites [i.e., (g/L ∙ 1,000)/molar mass] applied to the vector of concentrations per time points output of the model. The microbial group g/L concentration was first converted into bacteria/L by considering the weight of one bacterial cell of 4.6∙10^−12^ g (59, 60) and then transformed into pseudo-log_10_ (Log_10_) values considering as 1 bacterium/L every instance ≤0 bacterium/L (i.e., 0 g/L = 0Log_10_). Both metabolite mM and microbial group Log_10_ concentrations over time were compared to the observations collected through the *in vitro* experimentation calculating the RMSE via using the “rmse()” function of the Metrics package V0.1.4 (61) in R, which was also expressed as RMSE_%_ indicating the RMSE percentage of the observed mean [i.e., (RMSE/observed mean average) ∙ 100]. Moreover, regression analysis (LM) was carried out in R via the “lm()” function within the stats package, via implementing lm(modeled values ~ observed values).

### Continuous fermentation

During a parallel experiment, a 35-day continuous fermentation was carried out, in which a porcine fecal inoculum was grown under proximal colon conditions. The observations from this experiment were used to compare and parameterize the model. The fermentation was carried out in triplicate in a bespoke bioreactor/incubator composed of a main vessel (234 mL) connected via peristaltic pump to a flow-in vessel mimicking the ileal chyme inflow (Tables S21 and S22) and a flow out. The temperature was kept constant at 38°C, and anaerobiosis was maintained throughout the experiment, pH in the 6.0 ≤ pH ≤ 6.8 range and retention time of 9 hours, as found in the porcine proximal colon (48, 62, 63). The pooled fecal inoculum (60 g/L) was prepared in an anaerobic chamber (Whitley MG 500 + MG Airlock Anaerobic Workstation, Don Whitley Scientific Limited, UK) from naturally excreted feces, collected from five apparently healthy ~5-month-old male pigs fed on a standard commercial diet (SRUC Easter Howgate Farm). The inoculum was prepared in less than 3 hours from collection, and anaerobiosis was maintained throughout. In brief, 80 g of pooled fecal material was homogenized in 320 mL of medium and then sieved with a 100 µm cell strainer (Corning, UK). Media were sterilized by autoclave at 120°C for 15 minutes and then preincubated at 38°C while being connected to a CO_2_ canister through a polyvinylidene difluoride (PVDF) membrane filter (0.2 µm; Cole Parmer) to ensure anaerobiosis. Resazurin salt was also added to the medium to detect the eventual presence of oxygen, and 2 M NaOH was used to regulate the pH during the continuous fermentation. All the vessels of the reactor were connected to an anaerobic jar containing an anaerobic atmosphere generation bag (AnaeroGen 2.5 L,10269582 Thermo Fisher Scientific) and resazurin strips to detect the presence of O_2_. Samples collected at different time points through the fermentation were used to detect acetate, propionate, butyrate, and lactate concentrations via gas chromatography-mass spectrometry (GC-MS) to quantify microbial species through absolute qPCR and to qualitatively assess the population through 16S rRNA gene sequencing.

### Gas chromatography-mass spectrometry

Bioreactor samples were centrifuged at 14,000 rpm for 10 minutes at room temperature; thus, 2.5 µL of HCl were added to 100 µL of filtered supernatant (0.2 µm PVDF sterile 13 mm membrane filters, Whatman puradisc). SCFAs and lactate were extracted via adding 500 µL of diethyl ether (EE) spiked with 5 mM of the internal standard 2-ethylbutyric acid, vortexed for 15 minutes, and centrifuged for 3 minutes at 1,000× *g*. A total of 430 µL of this supernatant was stored in a fresh vial, while the pellet went through a second extraction with 500 µL of EE without internal standard. A total of 500 µL of pooled supernatant were transferred to the GC-MS instrument (GC/Q-TOF model 7200 A) and derivatized with 50 µL of N-tert-Butyldimethylsilyl-N-methyl trifluoroacetamide (Sigma-Aldrich, UK) with an automated protocol composed of: vortex for 15 minutes and incubation at room temperature for 15 minutes repeated twice (64) before the injection into the chromatography column (Agilent DB-5ms, 10 m guard + 30 m × 250 µm ID × 0.2 µm stationary phase).

GC-MS was carried on at the EdinOmics (Waddington Building, King’s buildings, The University of Edinburgh), with the following conditions: inlet temperature: 250°C, oven temperature 250°C–300°C; column: 70°C for 3 minutes; 20°C/minute up to 160°C; 30°C/minute up to 300°C and held for 3 minutes. The liner used for the injection of the sample was the ultra-inert liner universal, low PSI drop, and wool (5190–2295 Agilent). The mass spectrometry (GC/Q-TOF model 7200 A) source temperature was 230°C, quad temperature was 150°C, filament current was 35.10 µA, and emission current was 70 eV. Metabolite concentration was calculated from row data using the MassHunter Workstation Software, Quantitative Analysis, version B.07.01/Build 7.1.524.0 for GC-MS (Agilent Technologies, Inc. 2008).

### DNA isolation and 16S rRNA gene sequencing

A 250-µL aliquot of bioreactor samples or 250 µg of pooled feces were used to isolate total DNA using the QIAamp PowerFecal DNA Kit (QIAGEN, UK), following the manufacturer’s protocol. Library preparation was carried out via PCR (25 µL) prepared in a laminar flow PCR cabinet (Labcaire System, UK) and carried out in 25 µL containing 1× PCR buffer (NEB ThermoPol Buffer), 1 mM MgCl_2_, 200 µM of dNTP mix, 0.025 U/µL of Taq DNA polymerase (M0267S; New England BioLabs), 250 nM of both forward (a515F: 5′ GTGYCAGCMGCCGCGGTAA 3′) and reverse (806R: 5′ GGACTACHVGGGTWTCTAAT 3′) primers, 1 µL of template DNA, and nuclease-free PCR grade H_2_O. Conditions were 94°C for 3 minutes followed by 25 cycles at 94°C for 45 seconds, 50°C for 60 seconds, and 72°C for 90 seconds, finally 72°C for 10 minutes. The Wizard SV Gel and PCR Clean-Up System (Promega) were used to purify amplicons from 1.5% agarose gel, then quantified via Quant-iT PicoGreen dsDNA Assay Kit (Thermo Fisher Scientific) coupled with fluorescence measurement with excitation at 480 nm and emission at 520 nm (Spectra MAX GEMINI XS, Molecular Devices) allowing concentration normalization to 2.16 ng/µL in the single ready-to-sequence pool. A total of pooled 99 amplicon libraries were sequenced via Illumina MiSeq (MiSeq v2, 2 × 250 bp paired end reads, Illumina, UK) at Edinburgh Genomics (King’s Buildings, The University of Edinburgh), targeting the V4 region of the 16S rRNA gene, and amplified using Earth Microbiome Project primers, including 12 bp Golay barcode within the reverse primer (65–67).

### Bioinformatic analysis

A total of 11,066,443 FASTQ paired end demultiplexed reads (~110,000 sequences/sample) were imported as a “.qza” artifact in QIIME2 V2022.2 (68, 69) using the manifest method (69). A total of 88.8% of the reads were retained after using the “join-pairs” command of VSEARCH (70), with a resulting quality score of ~40 throughout their sequence length. Following, the quality-filtering was carried out with minimum Phred score of 20 (71, 72), and Deblur was used to denoise the filtered joined reads, which were first trimmed at 250 bp (73). The taxonomy was assigned using the q2-feature-classifier plugin, i.e., a Naïve Bayes classifier was trained based on the primers used and the last release of the Silva data base (132, 99% of similarities) (74–76).

### Bacterial absolute quantification

The V3 region of the 16S rRNA gene (Table 3) was targeted via qPCR to quantify the total number of bacteria through absolute quantification using an eight-point standard curve built using serial 10-fold dilution of a linearized plasmid containing the target gene as insert, while SYBR green was used as a detection chemistry, as previously described (77), allowing the conversion of the amplicon copy number per reaction, output of the qPCR, to bacterial concentration (number of bacteria per liter of sample).

**TABLE 3:** Primers used to amplify the V3 region of the 16S rRNA gene during qPCR

Target	Primer (5′→3′)	Annealing (°C)	Reference
V3	341F: CCTACGGGAGGCAGCAG	65	(78)
	518R: ATTACCGCGGCTGCTGG		

The relative abundance output of the 16S rRNA gene sequencing analysis was thus applied to the absolute bacterial concentration output of the qPCR, resulting in the g/L concentration of the modeled microbial groups. Therefore, concentration values were further transformed into pseudo-log_10_ as detailed for the modeled bacterial concentration allowing the comparison between observations and predictions.

## SUPPLEMENTAL MATERIAL

Tables S1 to S20 and Figures S1 to S17 msystems.00366-23-s0001.pdf Core genera data frame used to develop the core microbiota model.

Tables S21 and S22 msystems.00366-23-s0002.pdf Composition of the medium used during the continuous fermentation experiment.

ASM does not own the copyrights to Supplemental Material that may be linked to, or accessed through, an article. The authors have granted ASM a non-exclusive, world-wide license to publish the Supplemental Material files. Please contact the corresponding author directly for reuse.
