# Supplementary material for: Estimating the contribution of the porcine fecal core microbiota to metabolite production via mathematical modeling and in vitro fermentation
Source: mSystems. 2023 Dec 7;9(1):e00366-23. doi: 10.1128/msystems.00366-23 (PMC10805034; doi:10.1128/msystems.00366-23)
Supplement: Tables S1 to S20 and Figures S1 to S17 — Core genera data frame used to develop the core microbiota model. [file msystems.00366-23-s0001.pdf]

1 Introduction

MicroPop requires that the information regarding any of the modelled species is stored in data frame, which specify resource/metabolite stoichiometry and type, together with the values for the pH corners, maximum growth rate (μ_{max}), half-saturation constant (K) yield (Y) for each of the modelled metabolic pathways (1). In microPop, the resources are classified in essential (Se), substitutable (S), water-resource (Sw) and boosting (Sb). The latter describes any resource whose absence would still allow the bacterium to grow, although its presence would increase the grow rate according to a fraction of fermentation (Fb), going from 0 to 1, with 1 indicating total reliability on the Sb. The following sections describe in detail the data frames assembled to model the core genera and "*others*" (i.e., non-core genera), as specified in the main manuscript. A list of all the resources and metabolites modelled using microPop can be found in Table 1, whilst the metabolites and the resources in the below data frames are accompanied by stoichiometry in brackets and resources type. For each genus, optimal pH was modelled through the establishment of four pH corners (1), indicating two extreme pH values, above and below which no growth is possible, and two ideal pH values, within which growth is favoured.

Where possible, the data frames were based on observations from culture-based experiments of the representative species, whereas in five instances these were not available, and the data frames were built upon the available theoretical description of the representative genera/species. In all the cases, a simulation of the microbial dynamics based on the data frame of each genus was included, showing either the comparison with the observations used to inform the model or the main fermentation pattern of the genera with no available cultural information.

Table 1. List of resources and metabolites modelled and summarised in the microbial data frames.

Protein, non-starch polysaccharides (NSP), resistant starch (RS),Sugars, H_2, CO_2, CH_4, H_2O, Acetate, Propionate, Succinate, Hexose, Lactate, Formate, Ethanol, Butyrate, other

2 Prevotella

Prevotella is one of the most abundant genera in the porcine colon throughout pigs' life (2), with *P. ruminicola* being one of the most abundant species (3), which was therefore used as a representative species to model this genus. *P. ruminicola* grows both on carbohydrates and peptides but not amino acids (4), and the data frame was modelled through two different pathways, acknowledging the dynamics associated with either hexose or peptide fermentation (Table 2). Pathway 1 (carbohydrates) was based on observations from culture-based experiments of *P. ruminicola* growing on different resources (5). Whereas due to lack experimental evidence, pathway 2 (proteins) assumed the stoichiometry for protein degradation of *Bacteroides spp.*, belonging to the same order (1, 6). Assumption for pH corners was based on observations of *Bacteroides ruminicola* (basonym) growing at different pH values (7). A comparison between the modelled microbial dynamics of this genus and the observations used to inform its data frame is depicted in Figure 1.

Table 2. *Prevotella* data frame. Stoichiometry is depicted in brackets for both resources and metabolites, whilst resource type is indicated each time (*S*: substitutable; *Se*: essential; *Sb*: boosting; *Sw*: water).

	**1st pathway**	**2nd pathway**	**References**
**Resources**	NSP; *S* (6.319), RS; *S* (6.319); sugars (6. .319); H_2O, Sw (2)	Proteins; *Se* (6)	(1, 5, 6), this study (stoichiometry path. 1)
**Metabolites**	Acetate (6), lactate (1.5) succinate (4), propionate (1), , H_2 (0.3), CO_2 (3)	Acetate (2), propionate (1), succinate (1), H_2 (2), CO_2 (1), other (7)	
**μ_{max} (d^{-1})**	0.5 (NSP), 1(RS), 1 (sugars)	1 (protein)	(1, 6)
***K* (g/l)**	0.0025	0.0025	this study
***Y* (gl^{-1}/ gl^{-1})**	0.286 (NSP), 0.333 (RS), 0.333 (sugars)	0.2 (protein)	(1, 6, 8)
**pH corners**	5.25, 6.0, 6.5,7.0		(7)

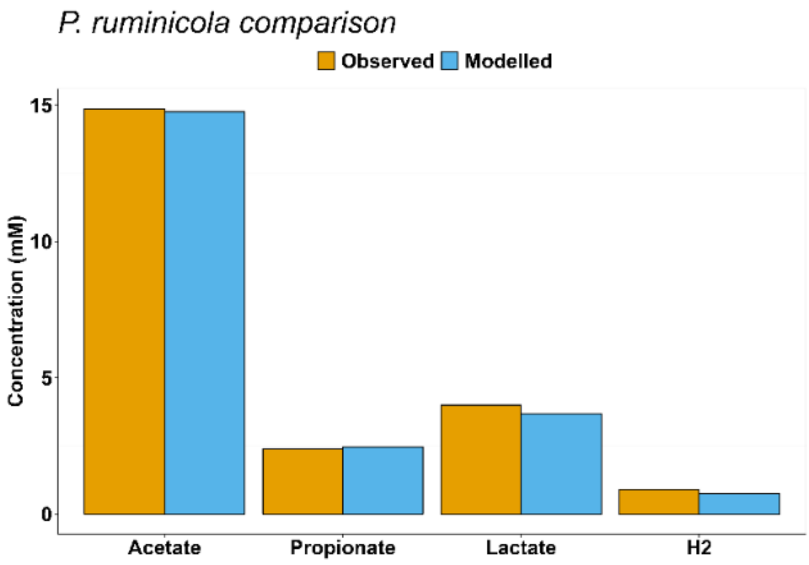

Figure 1. Comparison between the fermentation pattern of *P. ruminicola* AR29 growing in batch on pectin/glucose/arabinose and the output of the model simulating the same experimental conditions. The observations depicted here are the average SCFA values from either glucose, arabinose, or pectin 4g/l (5). Starting modelled conditions: NSP 2g/l, sugars 2 g/l, H_2O non limiting, *Prevotella* 0.1g/l.

3 Megasphaera

M. elsdenii, originally isolated from rumen, plays an important role in the pig gut (9, 10) and can grow on both carbohydrates and lactate (11) using two different pathways (6). Thus, this data frame was based on *M. elsdenii*, basing the assumption for the stoichiometry on batch culture experiments (9, 11) as shown in Table 3, whilst a comparison between the modelled microbial dynamics of this genus and the observations used to inform its data frame is depicted in Figure 2.

Table 3. *Megasphaera* data frame. Stoichiometry is depicted in brackets for both resources and metabolites, whilst resource type is indicated each time (*S*: substitutable; *Se*: essential; *Sb*: boosting; *Sw*: water).

	1st pathway	2nd pathway	Reference
**Resources**	NSP; *S* (1.574), RS; *S* (1.574), sugars; *S* (1.574)	Lactate; *Se* (0.416)	(6, 9, 11), stoichiometry: this study
**Metabolites**	Propionate (0.2), butyrate (0.2), acetate (0.35), CO_2 (5), H_2 (5)	Propionate (0.06), butyrate (0.06), acetate (0.08), CO_2 (0.5), H_2 (0.5)	
**μ_{max} (h^{-1})**	0.43 (NSP), 0.43 (RS), 1 (sugars) 0.43 (lactate)		(1, 6) this study (NSP, RS, lactate)
***K* (g/l)**	0.00045		this study
***y* (g/g)**	0.286 (NSP), 0.333 (RS), 0.333 (sugars), 0.11 (lactate)		(1, 6)
**pH corners**	4.6, 5.5, 6.05, 7.8		(9)

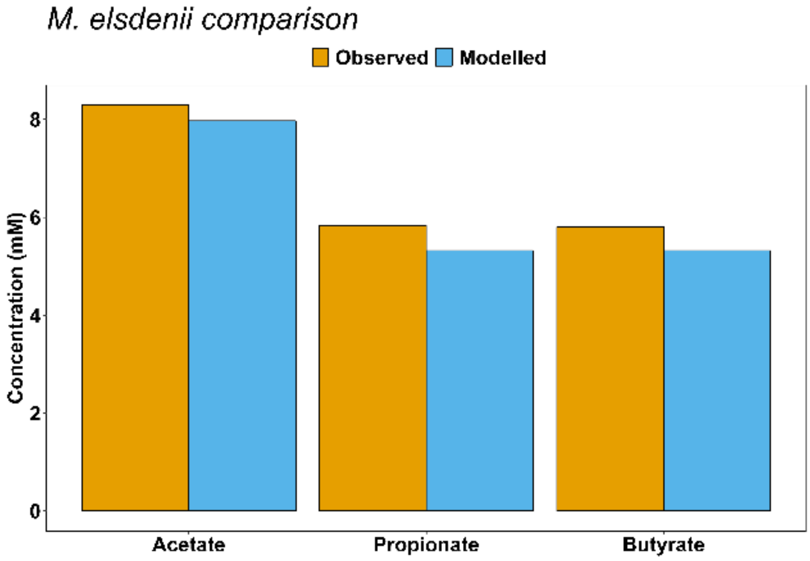

Figure 2. Comparison between the fermentation pattern of *M. elsdenii* growing in batch on sugars and lactate (11) and the output of the model simulating the same experimental conditions. Starting modelled conditions: sugars 4.5g/l, lactate 2.25g/l, *Megasphaera* 0.1g/l.

4 RC9

Due to lack of culture-based information, this data frame was modelled based on the theoretical description of the RC9 gut group, part of the *Rikenellaceae* family, and specifically on the characteristic of the two genera of this family, *Rikenella* and *Alistipses*, whose metabolism relies mainly on fermentation of few carbohydrates, mostly simple sugars, whilst producing mainly succinate, propionate and traces of acetate (12). The assumed dynamic parameters and the stoichiometry are depicted in Table 4, whilst the SCFA pattern output of the model based on this data frame is shown in Figure 3.

Table 4. RC9 data frame. Stoichiometry is depicted in brackets for both resources and metabolites, whilst resource type is indicated each time (*S*: substitutable; *Se*: essential; *Sb*: boosting; *Sw*: water).

	Pathway	Reference
**Resources**	Sugars; *Se* (1.3)	(12)
**Metabolites**	Propionate (1), succinate (1), acetate (0.1), H_2O (2)	
**μ_{max} (h^{-1})**	1 (sugars)	(1, 6)
***K* (g/l)**	0.001	this study
***y* (g/g)**	0.333 (sugars)	(1, 6)
**pH corners**	6, 6.5, 7, 8.5	(13, 14)

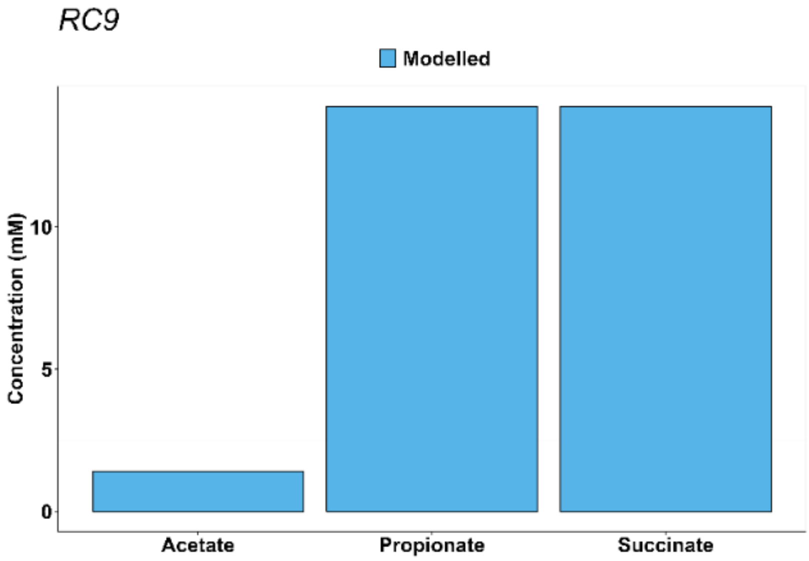

Figure 3. Predicted fermentation pattern of RC9, based on the theoretical description of *Rikenella* and *Alistipses*, whose metabolism relies mainly on fermentation of mostly simple sugars, whilst producing mainly succinate, propionate and trace of acetate (12)

5 Streptococcus

The data frame for *Streptococcus* was based on *S. hyointestinalis* and *S. bovis*. The former is commonly found in the pig intestine (15), whose acid production is reported from glucose, fructose, mannose, galactose, lactose, N-acetylglucosamine, arbutin, salicin, maltose, saccharose, trehalose, and starch (16). The stoichiometry was based on the heterofermentative metabolism (Table 5) following the genus description, according to which all the belonging species ferment carbohydrates, producing predominantly lactic acid although minor amounts of acetic, formate, ethanol, and CO_2 may also be found. (17). *S. bovis* was considered as the representative species for this fermentative pathway (18) and therefore the model output was compared to the observed SCFA production as reported for this species (19) as depicted in Figure 4.

Table 5. *Streptococcus* data frame. Stoichiometry is depicted in brackets for both resources and metabolites, whilst resource type is indicated each time (*S*: substitutable; *Se*: essential; *Sb*: boosting; *Sw*: water).

	**Pathway**	**Reference**
**Resources**	RS; *S* (4), sugars; *S* (4), H_2O;*Sw* (1)	(16, 17)
**Metabolites**	Lactate (6), acetate (1), formate (1), ethanol (1), CO_2 (1) and H_2 (1)	
μ_{max} **(h^{-1})**	0.25 (RS), 1 (sugars)	(1, 6)
***K* (g/l)**	0.001	this study
***y* (g/g)**	0.333 (RS), 0.333 (sugars)	(1, 6)
**pH corners**	5, 5.5, 6.5, 7	(20)

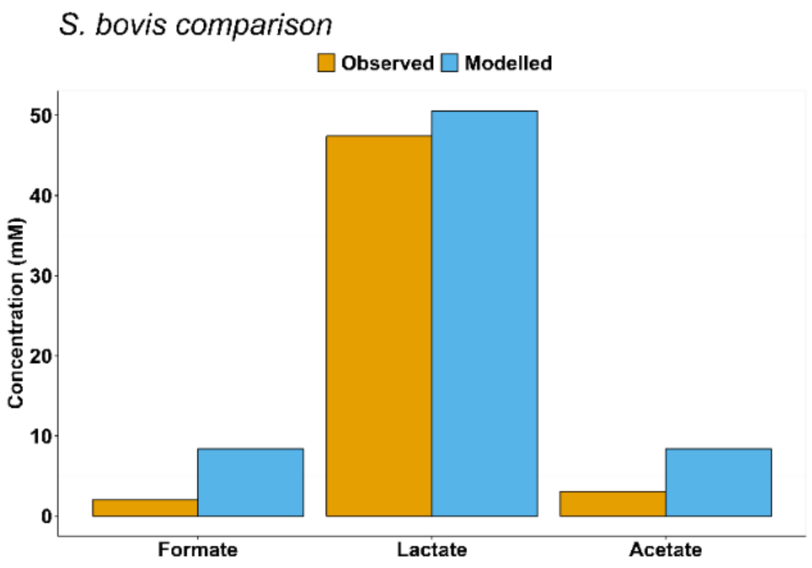

Figure 4. Comparison between the fermentation pattern of *S. bovis* growing in batch on glucose (19) and the output of the model simulating the same experimental conditions. Starting modelled conditions: sugars 9g/l H_2O non limiting *Streptococcus* 0.1g/l.

6 Lactobacillus

Lactobacillus species are frequently reported in the pig intestine, such as for *L. mucosae* producing both D- and L-lactate from non-complex saccharides (21), *L. pontis* described to produce lactate, acetate, ethanol and CO_2 from fermentation of maltose or fructose (22), and for *L. reuteri* , which is found as endogenous in pigs (23). This data frame was based on the heterofermentative metabolism of *L. reuteri* (Table 6), and a comparison between *L. reuteri* strains DSM20016 and DSM17938 (24), i.e., with similar dynamics and metabolite pattern) and the output of the model simulating the same experimental conditions are shown in Figure 5. Acetate production was not reported for *L. reuteri* (24), however it was modelled here as mentioned by other authors for other *Lactobacillus* species (25).

Table 6. *Lactobacillus* data frame. Stoichiometry is depicted in brackets for both resources and metabolites, whilst resource type is indicated each time (*S*: substitutable; *Se*: essential; *Sb*: boosting; *Sw*: water).

	**Pathway**	**Reference**
**Resources**	Sugars; S (7)	(24, 26, 27)
**Metabolites**	Acetate (1) Lactate (9) Ethanol (5), CO_2 (3.635)	
**μ_{max} (h^{-1})**	1	(1, 6)
***K* (g/l)**	0.001	this study
***y* (g/g)**	0.333	(1, 6)
**pH corners**	4, 6, 6.8, 7.5	(28, 29)

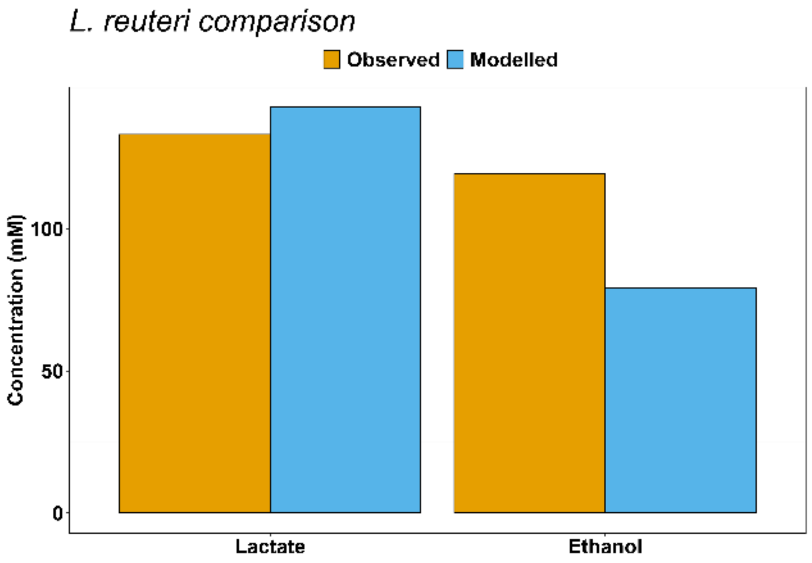

Figure 5. Comparison between the fermentation pattern of *L. reuteri* growing in batch for 24 hours (24) and the output of the model simulating the same experimental conditions. Observed metabolite concentration is the extrapolated average between the two strains (DSM20016 and DSM17938) as shown in Fig.4 of the original manuscript, i.e., ~12g/l for lactate and ~5.5g/l for ethanol, respectively. Starting modelled conditions: sugars 30g/l, *Lactobacillus* 0.1g/l.

7 Alloprevotella

Alloprevotella spp. are moderately saccharolytic bacteria, whose main products of fermentation are mainly acetate and succinate (30). This data frame (Table 7) is based on the description of the basonym *P. tannerae*, consequently reclassified as *A. tannerae* (30, 31) and a representation of the SCFA pattern output of the model is depicted in Figure 6.

Table 7. *Alloprevotella* data frame. Stoichiometry is depicted in brackets for both resources and metabolites, whilst resource type is indicated each time (*S*: substitutable; *Se*: essential; *Sb*: boosting; *Sw*: water).

	Pathway	Reference
**Resources**	RS; *S* (1) Sugars; *S* (1), CO_2; *Se* (1)	(30)
**Metabolites**	Succinate (1), acetate (1), formate (1)	
μ_{max} **(h^{-1})**	1 (RS), 1 (sugars)	(1, 6)
***K* (g/l)**	0.002	this study
***y* (g/g)**	0.333 (RS); 0.333 (sugars)	(1, 6)
**pH corners**	5, 6.5, 7, 8	(30)

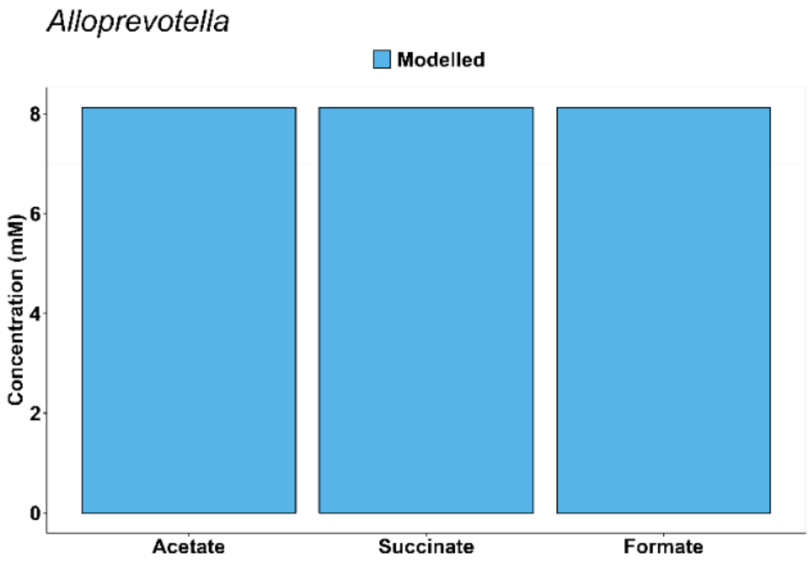

Figure 6. Predicted batch fermentation pattern of *Alloprevotella*, based on the theoretical description of *P. tannerae* isolated from human oral cavity (consequently reclassified as *A. tannerae*) (30, 31). Starting concentration: sugars 1g/l, RS 1g/l CO_2 non limiting, *Alloprevotella* 0.1g/l

8 Clostridium

C. butyricum was chosen as a reference species for this data frame (Table 8), due to its documented presence and correlation to good health in pigs (32), and the assumption for its stoichiometry was based on the butyrate-kinase pathway (33). This species can ferment most carbohydrates, including RS and some NSP (e.g., pectin), whilst mainly producing butyrate, acetate, formate, with minor amount of lactate and succinate, H_2 and CO_2 (34). The data frame was developed in two pathways, with the first one based on cultural-based observations (35), the second pathway was based on the theoretical description of lactate conversion to butyrate, when in presence of acetate (36). The model somewhat underestimated acetate and overestimated lactate production (Figure 7), when compared to SCFA patterns from glucose fermentation (35), however the chosen stoichiometry was thought to be representative of the genus description as described by other studies (36).

Table 8. *Clostridium* data frame. Stoichiometry is depicted in brackets for both resources and metabolites, whilst resource type is indicated each time (*S*: substitutable; *Se*: essential; *Sb*: boosting; *Sw*: water).

	**1st pathway**	**2nd pathway**	**Reference**
**Resources**	Sugars; *S* (4.7215), RS; *S* (4.7215), NSP; *S* (4.71)	Lactate: *Se* (4), acetate: *Se* (2)	(6, 34-36)
**Metabolites**	Acetate (1.8), lactate (1.5), butyrate (2), formate (2), ethanol (0.2), succinate (0.18), CO_2 (7), H_2 (0.001).	Butyrate (3), CO_2 (4), H_2 (2), H_2O (2)	
**μ_{max} (h^{-1})**	0.3 (NSP), 0.3 (RS), 1 (sugars), 0.0006 (lactate)		(1, 6), lactate: this study
***K* (g/l)**	0.001		this study
***y* (g/g)**	0.286 (NSP), 0.333 (RS), 0.333 (sugars), 0.11 (lactate)		(1, 6)
**pH corners**	6.0, 6.5, 6.6, 7.5		(37)

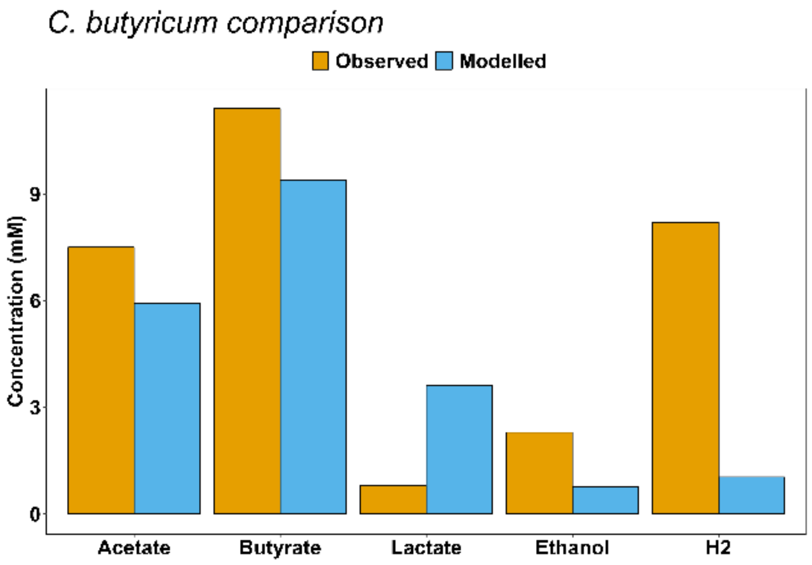

Figure 7. Comparison between the fermentation pattern of *C. butyricum* growing in batch on glucose (35) and the output of the model simulating the same experimental conditions. Starting modelled conditions: sugars 5g/l, *Clostridium* 0.1g/l.

9 Treponema

No culture-based information was found for *T. berlinense* and *T. porcinum*, isolated from the swine gut (38), thus the stoichiometry assumption for this data frame (Table 9) was based on the metabolisms of *T. succinifaciens*, isolated from colon of pigs (39). *T. succinifaciens* ferments carbohydrates but not protein and requires CO_2 for growth, whilst main metabolites produced are acetate, formate, succinate and lactate (39) and a comparison with culture-based data is depicted in Figure 8. pH corners were assigned following theoretical considerations of this species growing in the pig colon.

Table 9. *Treponema* data frame. Stoichiometry is depicted in brackets for both resources and metabolites, whilst resource type is indicated each time (*S*: substitutable; *Se*: essential; *Sb*: boosting; *Sw*: water).

	**Pathway**	**Reference**
**Resources**	RS; *S* (1.2), sugars; *S* (1.2), CO_2; *Se* (0.51)	(39)
**Metabolites**	Acetate (1.3), formate (1.21), succinate (0.58) and lactate (0.405)	
**μ_{max} (h^{-1})**	0.5 (RS), 1 (sugars)	this study
***K* (g/l)**	0.001	
***y* (g/g)**	0.333 (RS), 0.333 (sugars)	(1, 6)
**pH corners**	6, 6.2, 6.5, 7	this study

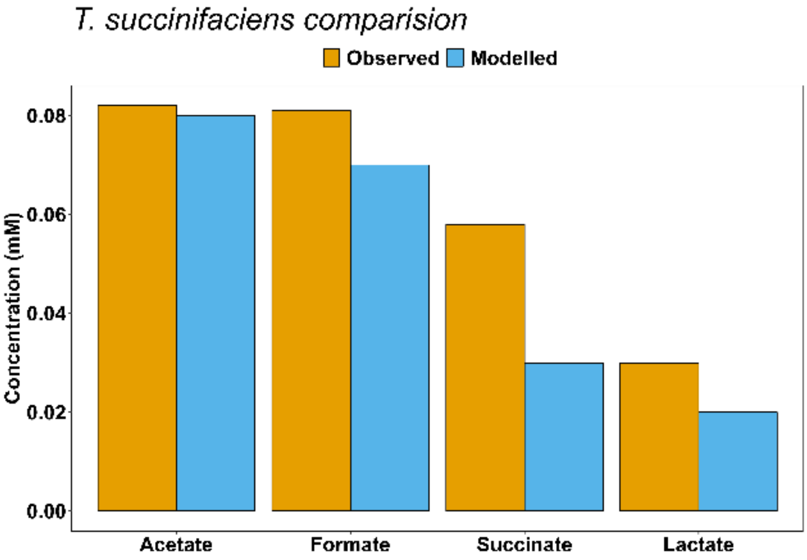

Figure 8. Comparison between the fermentation pattern of *T. succinifaciens* growing in batch on glucose (39) and the output of the model simulating the same experimental conditions. Starting modelled conditions: sugars 18.016mg/l, CO_2 2.26mg/l *Treponema* 0.1g/l.

10 Faecalibacterium

Faecalibacterium prausnitzii, the reference species of this data frame, produces butyrate, lactate, formate and CO_2, through net consumption of acetate as a boosting resource (40, 41). The data frame (Table 10) was parametrised based on a batch culture-based experiment of *F. prausnitzii* strain A2-165 growing in YCFAG medium containing glucose and acetate (42). A comparison between these observations and the modelled output is depicted in Figure 9.

Table 10. *Faecalibacterium* data frame. Stoichiometry is depicted in brackets for both resources and metabolites, whilst resource type is indicated each time (*S*: substitutable; *Se*: essential; *Sb*: boosting; *Sw*: water, Fb: fraction of fermentation for Sb).

	**Pathway**	**Reference**
**Resources**	NSP; S (11.456), RS; S (11.456), sugars; S (11.456), acetate; Sb (Fb=0.1) (4)	(6, 41)
**Metabolites**	Butyrate (15), lactate (1), formate (14), CO_2 (4), H_2O (4)	
**μ_{max} (h^{-1})**	0.6 (NSP), 0.3 (RS), 1 (sugars)	(1, 6)
***K* (g/l)**	0.005	this study
***y* (g/g)**	0.286 (NSP), 0.333 (RS), 0.333 (sugars)	(1, 6)
**pH corners**	4.6, 5.7, 6.7, 7	(43, 44)

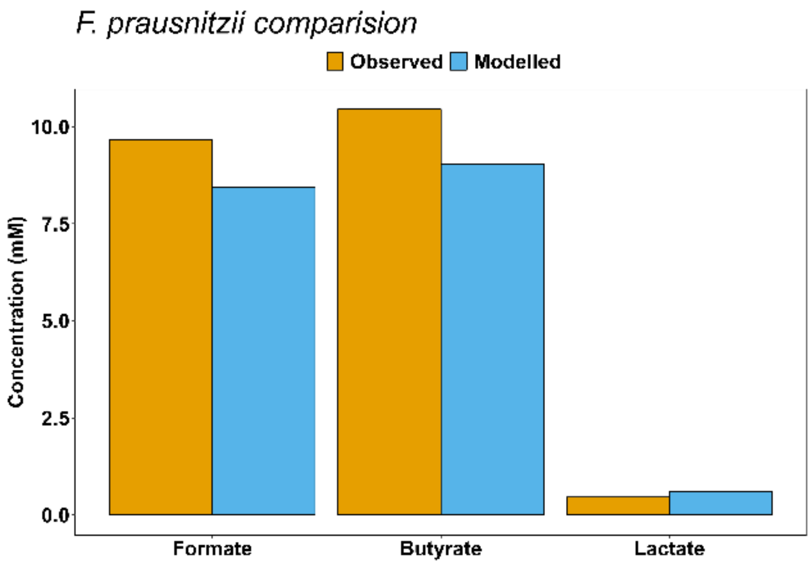

Figure 9 Comparison between *F. prausnitzii* A2-165 observations from growth in YCFAG medium containing glucose (10mM) and acetate (33mM) for 24 hours (42) and predictions generated modelling the same starting conditions (*Faecalibacterium:* 0.1g/l starting conditions).

11 Succinivibrio

S. dextrinosolvens was chosen as a reference species for this data frame (Table 11) . *S. dextrinosolvens* are strictly anaerobic, Gram-negative rods, whose metabolism relies mainly on carbohydrate fermentation, with glucose, D-xylose, maltose, galactose, dextrin and pectin being the main resource used, and whilst some strains use cellobiose as a resource none of them seem to be able to use starch directly (45–47). Stoichiometry assumption was based on the metabolite pattern reported for *S. dextrinosolvens* growing in rumen fluid-glucose medium (45, 46), and a comparison between these observations and the modelled output is depicted in Figure 10.

Table 11. *Succinivibrio* data frame. Stoichiometry is depicted in brackets for both resources and metabolites, whilst resource type is indicated each time (*S*: substitutable; *Se*: essential; *Sb*: boosting; *Sw*: water).

	Pathway	Reference
**Resources**	Sugars; *S* (4.374), NSP; *S* (4.374),CO_2; *Se* (4)	
**Metabolites**	Succinate (3.5), acetate (3.2), formate (2.7), lactate (0.6) and H_2O (10)	(45, 46)
**μ_{max} (h^{-1})**	0.7 (NSP), 1 (sugars)	(1, 6)
***K* (g/l)**	0.00012	this study
***y* (g/g)**	0.286 (NSP), 0.333 (sugars)	(1, 6)
**pH corners**	6, 6.8, 6.9, 8	(48)

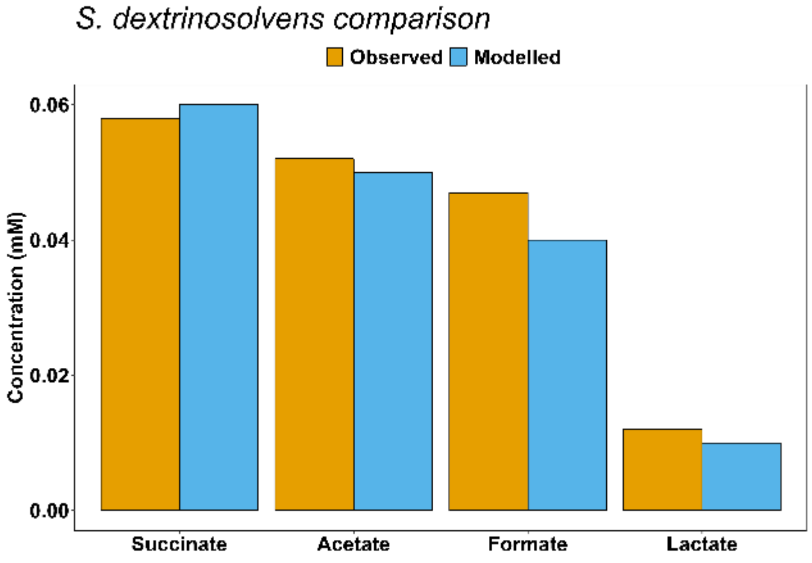

s

Figure 10. Comparison between the fermentation balance showed for *S. dextrinosolvens* growing in batch on glucose (46) and the output of the model simulating the same experimental conditions. Starting conditions: sugars 0.018g/l, CO_2 0.04g/l assumed from CO_2 consumption stated in the manuscript and *Succinivibrio* 0.1g/l.

12 Blautia

Blautia data frame was developed through three pathways (Table 12) based on the genus description, and representative of *Blautia* growth on carbohydrates, CO_2 and H_2 and formate, respectively (6, 49–52). A comparison between the modelled fermentation pattern and the observations of of *B. hydrogenotrophica* growing on fructose and formate is depicted in Figure 11.

Table 12. *Blautia* data frame. Stoichiometry is depicted in brackets for both resources and metabolites, whilst resource type is indicated each time (*S*: substitutable; *Se*: essential; *Sb*: boosting; *Sw*: water).

	1st pathway	2nd pathway	3rd pathway	References
**Resources**	NSP; *S* (1.033), RS; *S* (1.033), sugars; *S* (1.033)	CO_2; *Se* (0.1), H_2; *Se* (0.1), sugars (0.6)	Formate; *Se* (1.87), NSP; *S* (1), RS; *S* (1), sugars; *S* (1)	(1, 6, 49, 53-55)
**Metabolites**	Acetate (1.6), lactate (1)	Acetate (0.25), H_2O (5.29)	Acetate (0.5), H_2 (8), CO_2 (5)	
**μ_{max} (d^{-1})**	0.3 (NSP), 0.3 (RS), 1 (sugars)	0.1 (CO_2), 0.1 (H_2), 0 (sugars)	0.3 (NSP), 0.3 (RS), 1 (sugars)	(1, 6)
***K* (g/l)**		0.00011		this study
***Y* (gl^{-1}/ gl^{-1})**	0.286 (NSP), 0.333 (RS), 0.333 (sugars)	0.03 (CO_2), 0.03 (H_2)	0.286 (NSP), 0.333 (RS), 0.333 (sugars)	(1, 6)
**pH corners**		6, 6.5, 6.6, 7		(56, 57)

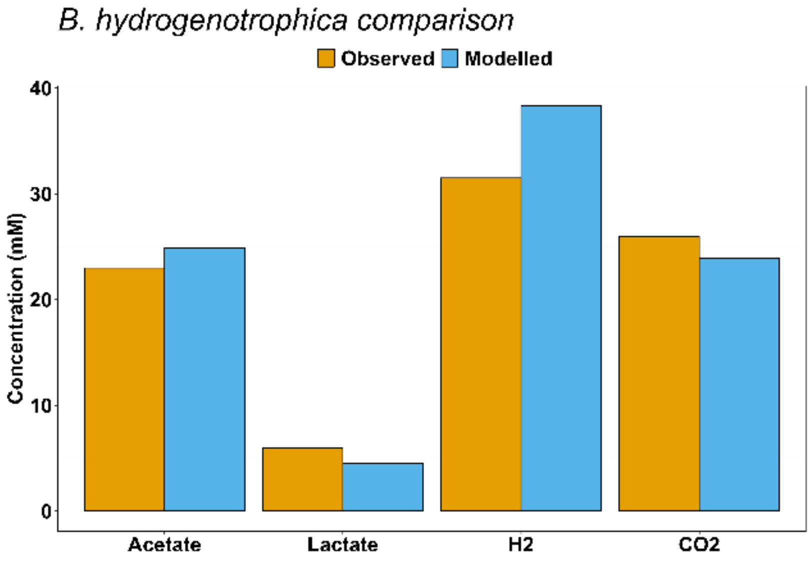

Figure 11. Comparison between .*B. hydrogenotrophica* growing on fructose glucose and formate (58) and the output of the model simulating the same experimental conditions. Starting modelled conditions: sugars 9.008 g/l, formate 2.3g/l, *Blautia* 0.1g/l and pH 6.8.

13 Phascolarctobacterium

At the time of writing, three *Phascolarctobacterium* species were described, *P. faecium* (59), *P. succinatutens* (60), and *P. wakonense* (61), isolated from koala faeces, human faeces and marmoset faeces, respectively. Unfortunately, no cultural-based data were found, therefore this frame (Table 13) was developed through assumptions based on genus description, which typically consume succinate producing propionate (59). The stoichiometry was based on the formulae 2Succinate ($C_4H_6O_4$) → 2Propionate ($C_3H_6O_2$) + CO_2 (Figure 12). Assumption for μ_{max} was based on bacterial growth on organic acids, whilst assumption for *Y* was based on theoretical considerations regarding ATP production per consumption of succinate (1, 6). Assumption for pH corners was based on theoretical considerations of the presence of this genus in the gastrointestinal tract of pigs.

Table 13.*Phascolarctobacterium* data frame. Stoichiometry is depicted in brackets for both resources and metabolites, whilst resource type is indicated each time (*S*: substitutable; *Se*: essential; *Sb*: boosting; *Sw*: water).

	Pathway	Reference
**Resources**	Succinate; *Se* (2.255)	(59)
**Metabolites**	Propionate (3) and CO_2 (1)	
**μ_{max} (h^{-1})**	0.2	(1, 6)
***K* (g/l)**	0.01	this study
***y* (g/g)**	0.08	(1, 6)
**pH corners**	6, 6.5, 7, 8	based on theorical considerations

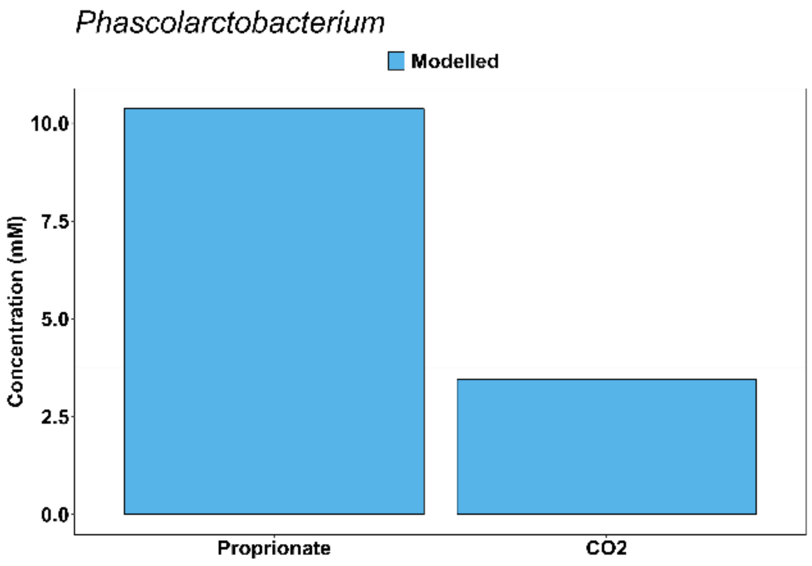

Figure 12. Predicted fermentation pattern of *Phascolarctobacterium*, based on the theoretical description of the genus.

14 Ruminococcus

The data frame for *Ruminococcus* was summarised the metabolism of the 13 described species of this genus (62) through two pathways (Table 14). The assumption for stoichiometry for the first pathway was based on continuous monocultures of *R. bromii*, originally isolated from human faeces growing on 0.5% starch (63). The assumption for stoichiometry for the second pathway was based on observations of *R. flavefaciens* isolated from human colon growing in pure culture (6, 64, 65). The model was tested towards *in vitro* continuous monoculture observations of resistant starch fermentation by *Ruminococcus bromii* (63) and a comparison between the modelled and observed outputs are depicted in Figure 13.

Table 14. *Ruminococcus* data frame. Stoichiometry is depicted in brackets for both resources and metabolites, whilst resource type is indicated each time (*S*: substitutable; *Se*: essential; *Sb*: boosting; *Sw*: water).

	1st pathway	2nd pathway	Reference
**Resources**	NSP; *S* (0.848), RS; *S* (0.848)	NSP; *S* (1.134), RS; *S* (1.134), H_2O; *Sw* (1)	(63, 64)
**Metabolites**	Acetate (0.5), succinate (1), lactate (0.05), H_2 (0.1)	Acetate (0.7), formate (0.7), ethanol (1.2), lactate (0.05) CO_2 (2), H_2 (0.1)	
**μ_{max} (h^{-1})**	0.7 (NSP), 0.15 (RS)	0.15 (NSP), 0.6 (RS)	(1, 6)
***K* (g/l)**	0.0004	0.00042	this study
***y* (g/g)**	0.286 (NSP), 0.333 (RS)		(1, 6)
**pH corners**	6.0, 6.25, 6.5, 7.0		(7)

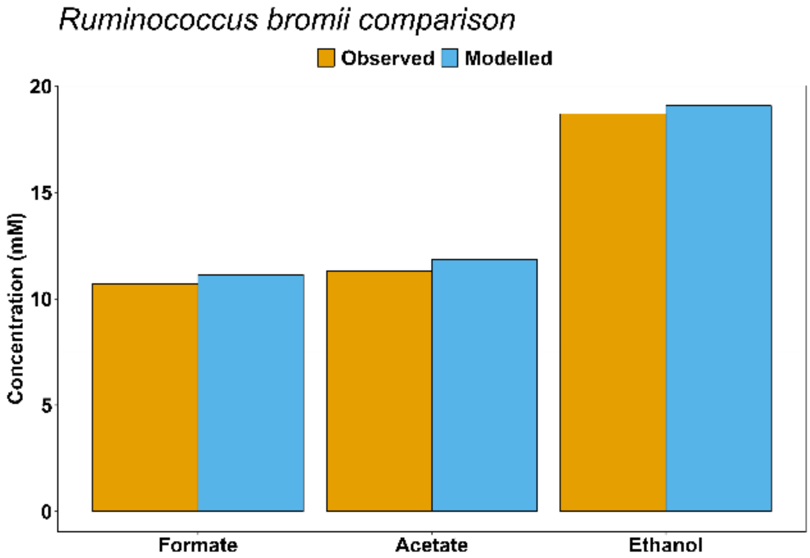

Figure 13. Comparison between the fermentation pattern of continuous monoculture (100 hours) sof *Ruminococcus bromii* growing on 0.5% RS (63) and the output of the model simulating the same experimental conditions. Starting modelled conditions: RS 5 g/l, *Ruminococcus* 0.1g/l, dilution rate (D) 0.042h^{-1} (calculated from flow out of 250ml/d and main vessel volume of 250ml).

15 Parabacteroides

Amongst the fifteen species of *Parabacteroides* genus described at the time of writing, 11 have been isolated from human intestinal or faecal samples, and whilst none of them have been directly isolated from the porcine gut (Table 15) their presence has been documented within the pig microbiota (66).

As depicted in **Error! Reference source not found.**, due to the absence of culture-based observation for this genus, the data frame and the stoichiometry assumption were based on the succinate pathway described for this genus (67), distinguishing two pathways, for hexose and protein degradation, respectively. Modelling parameter values were based on the taxonomically closely related *Bacteroides spp* as in the human model (1, 6), and the pH corner assumption was based on *P. acidifaciens* (68).

Table 15. List of classified *Parabacteroides* species described at the time of writing.

#	Species	Isolated from	Reference
1	*Parabacteroides acidifaciens*	Human faeces	(68)
2	*Parabacteroides bouchesdurhonensis*	Human faeces	(69)
3	*Parabacteroides chartae*	Paper mill wastewater	(70)
4	*Parabacteroides chinchilla*	Chinchilla (faeces	(71)
5	*Parabacteroides chongii*	Human blood	(72)
6	*Parabacteroides distasonis*	Human faeces	(67)
7	*Parabacteroides faecis*	Human faeces	(73)
8	*Parabacteroides goldsteinii*	Human Intestine	(74)
9	*Parabacteroides gordonii*	Human blood	(75)
10	*Parabacteroides johnsonii*	Human faeces	(76)
11	*Parabacteroides massiliensis*	Human faeces	(77)
12	*Parabacteroides merdae*	Human faeces	(78)
13	*Parabacteroides pacaensis*	Human gut	(79)
14	*Parabacteroides provencensis*	Human gut	(79)
15	*Parabacteroides timonensis*	Human faeces	(80)

Table 16. Parabacteroides BU data frame. Stoichiometry is depicted in brackets for both resources and metabolites, whilst resource type is indicated each time (*S*: substitutable; *Se*: essential; *Sb*: boosting; *Sw*: water).

	1st pathway	2nd pathway	Reference
**Resource**	Sugars; *S* (1.918), NSP; *S* (1.918) and RS; *S* (1.918)	Protein; *Se* (3.112)	(6, 65, 67)
**Metabolites**	Succinate (1), acetate (2) propionate (0.8), H_2 (2) and CO_2 (1)	Acetate (2), propionate (0.8), succinate (1) H_2 (2) and CO_2 (1)	
**μ_{max} (h^{-1})**	0.5 (NSP); 1 (RS); 1 (Sugars); 1 (Protein)		(6, 65)
***K* (g/l)**	0.005		this study
***y* (g/g)**	0.286 (NSP), 0.333 (RS), 0.333 (Sugars), 0.200 (Protein)		(6, 65)
pH corners	6.0, 6.5, 7.5,10		(68)

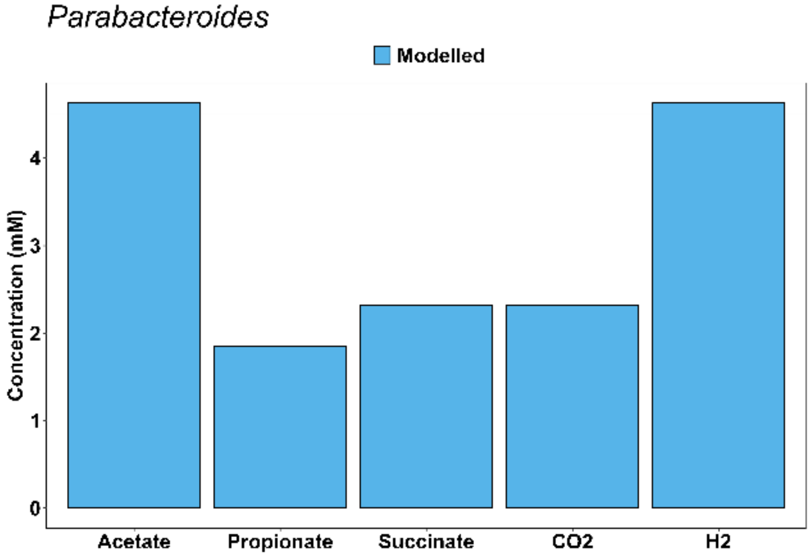

Figure 14. Predicted fermentation pattern of *Parabacteroides*, based on the theoretical description of the genus (67).

16 Escherichia

Some of the *E. coli* strains have been classified as commensals in pigs (81), although they can be cause of colibacillosis for different aetiology, such as waning-driven microbial stress (82, 83). This data frame (Table 17) was based on the fermentative metabolism of the facultative anaerobe *Escherichia coli*, which in absence of oxygen can ferment sugars through mixed acid fermentation (84). The reported fermentation balance (85) and observations from batch cultures (86) were used to derive the stoichiometry. The assumption for μ_{max} was based on batch culture observations of strain ATCC11303 growing on glucose (87), whilst yield and *K* followed the same theoretical and empirical considerations previously discussed (1).

Observations for *E. coli* growing on xylose (1mM) at pH 6.5 (86) were simulated using *Escherichia* BU data frame and a comparison between observation and predictions is depicted in **Error! Reference source not found.** (starting conditions were0.18g/l of sugars, pH 6.5, whilst the model simulated the batch culture for 24 hours).

Table 17. *Escherichia* BU data frame. Stoichiometry is depicted in brackets for both resources and metabolites, whilst resource type is indicated each time (*S*: substitutable; *Se*: essential; *Sb*: boosting; *Sw*: water).

	**Metabolic pathway**	**Reference**
**Resources**	Sugars; *S* (0.873)	(85, 86)
**Metabolites**	Acetate (0.47), ethanol (0.31), lactate (0.73), formate (0.91), succinate (0.06)	
**μ_{max} (h^{-1})**	0.65	(87)
***K* (g/l)**	0.001	this study
***y* (g/g)**	0.333 (sugars)	(1, 6)
**pH corners**	5.5, 6, 7.5, 8	(88)

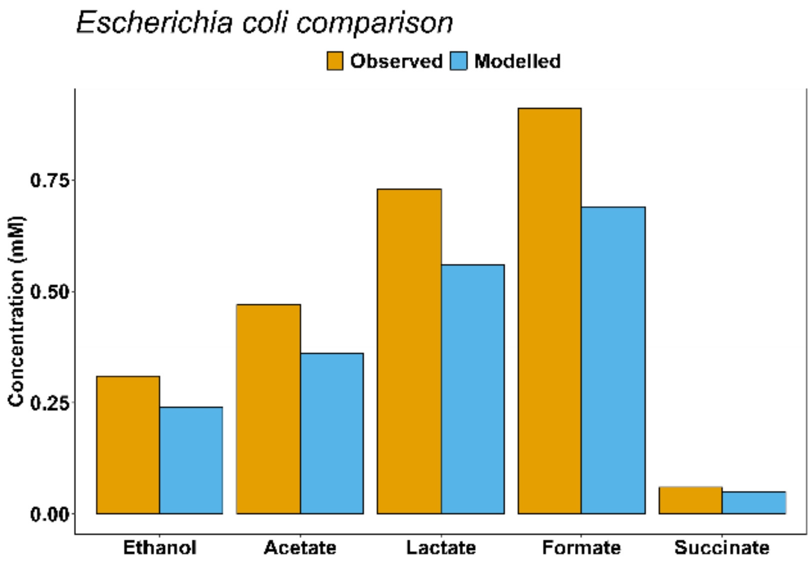

Figure 15. Comparison between *E. coli* growing on xylose (86) and the output of the model simulating the same experimental conditions. Starting modelled conditions: sugars 0.18g/l, pH 6.5, *Escherichia* 0.1g/l.

17 Turicibacter

This data frame was based on the genus description, with reference to the type species, *T. sanguinis*, able to grow on meat-chopped carbohydrates medium using proteins and few sugars only (89, 90). The two pathways in the data frame (Table 18) were based on the pyruvate formate lyase and the lactate dehydrogenase pathways (89, 90). Formate production was not reported for the type species, therefore it was assumed that the latter was completely converted into H_2 and CO_2, modelling the reaction catalysed by the formic hydrogen-lyase as a part of the pyruvate formate lyase pathway. The modelling parameter values were based on theoretical considerations, in a similar fashion as previously carried out (1, 6).

Table 18. *Turicibacter* data frame. Stoichiometry is depicted in brackets for both resources and metabolites, whilst resource type is indicated each time (*S*: substitutable; *Se*: essential; *Sb*: boosting; *Sw*: water).

	1st pathway	2nd pathway	References
Resources	Sugars; *S* (2), H_2O; *Sw* (1)	Protein; *S* (3.246), H_2O; *Sw* (1)	(89, 90)
Metabolites	Lactate (3), Acetate (1), H_2 (2),CO_2 (1)	Lactate (3), Acetate (1), H_2 (2),CO_2 (1)	
μ_{max} (h^{-1})	1 (sugars), 1 (protein)		(1, 6)
K (g/l)	0.001		this study
y (g/g)	0.333 (sugars), 0.2 (protein)		(1, 6)
pH corners	6.4, 7.0 ,7.5, 8.1		(89, 90)

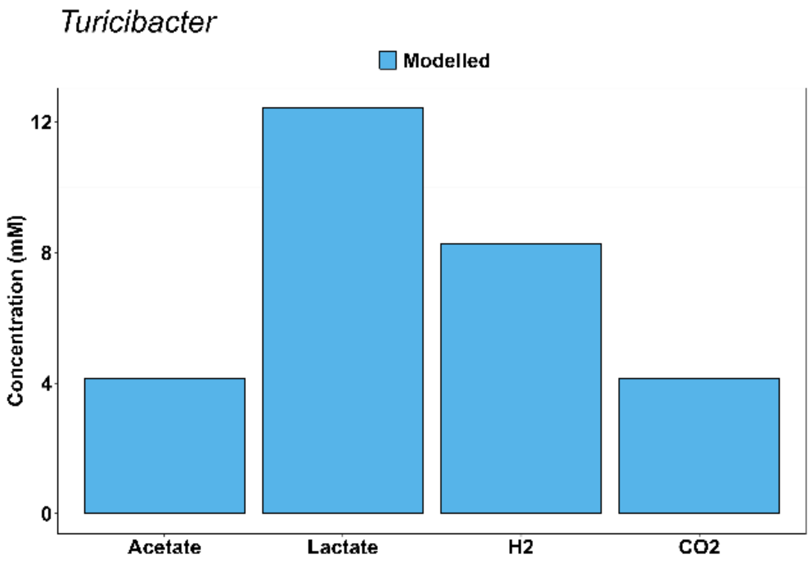

Figure 16. Predicted fermentation pattern of *Turicibacter* spp., based on the theoretical description of *T. sanguinis*, with metabolism based on pyruvate formate lyase and the lactate dehydrogenase pathways, whilst formate is entirely transformed in H_2 and CO_2 (90).

18 Others

The group *others* was used to model the rest of the microbiota members that were not considered as core genera. The modelling approach in this case was similar to the rest of the groups described above, as all the composing pathways were organised in a data frame, including stoichiometry, dynamic parameters and pH corners. However, *others* was composed by a higher number of metabolic pathways (i.e., 16 in total), reflective of the microbiota summarised in 10 different microbial functional groups (MFG), following a previous modelling approach based on such functional classification (1, 6). A summary and a brief description of the 10 MFGs modelled in this data frame is depicted in Table 19, whereas the 16 pathways composing the data frame are described in detail in Table 20. The values for *y* followed the same theoretical considerations as per original manuscript (1), whereas the rest of the dynamic parameters were derived through reverse modelling via comparison to the bioreactor experiment as detailed in the main manuscript. The pH corner were modelled as the average of all pH corners from the rest of the MFGs described by Kettle et al., (2018), i.e., 5.08, 5.83, 7.33, 8.08.

Table 19. MFGs modelled through the 16 pathways of the *others* data frame.

MFG	Description
1	Acetate-propionate-succinate group
2	Non-butyrate forming starch degraders
3	Non-butyrate forming fibre degraders
4	Lactate producers
5	Butyrate producers (1), produce principally butyrate H_2 and CO_2
6	Butyrate producers (2), produce butyrate, lactate formate and CO_2
7	Propionate producers
8	Butyrate producers (3), able to utilise lactate
9	Acetogens
10	Methanogens

Table 20. *Others* data frame composed of 16 pathways derived by the relative MFGs (1).

MFG1										
**Pathway**	1					2				
	Type	*K*	*y*	μ_{max}	stoichiometry	Type	*K*	*y*	μ_{max}	stoichiometry
**units**	none	g/L	g/g	/h	mol	none	g/L	g/g	/h	mol
**Protein**						S	0.01	0.2	0.5	6
**NSP**	S	0.01	0.286	1	2					
**RS**	S	0.01	0.333	1	2					
**Sugars**										
**H2**	P				2	P				2
**CO2**	P				1	P				1
**CH4**										
**H2O**										
**Acetate**	P				2	P				2
**Propionate**	P				1	P				1
**Succinate**	P				1	P				1
**Hexose**										

**Lactate**										
**Formate**										
**Ethanol**										
**Butyrate**										
**other**						P				7

**MFG2**					
**Pathway**	3				
	Type	*K*	*y*	μ_{max}	stoichiometry
**units**	none	g/L	g/g	/h	mol
**Protein**					
**NSP**	S	0.01	0.002	0.01047	1
**RS**	S	0.01	0.006	0.01047	1
**Sugars**					
**H2**	P				4
**CO2**	P				2
**CH4**					
**H2O**	Sw				2
**Acetate**	P				2

**Propionate**					
**Succinate**					
**Hexose**					
**Lactate**					
**Formate**					
**Ethanol**					
**Butyrate**					
**other**					

**MFG3**					
**Pathway**	4				
	Type	*K*	*y*	μ_{max}	stoichiometry
**units**	none	g/L	g/g	/h	mol
**Protein**					
**NSP**	S	0.01	0.286	0.156	1
**RS**	S	0.01	0.333	0.033	1
**Sugars**					
**H2**	P				1
**CO2**					
**CH4**					

**H2O**					
**Acetate**	P				1
**Propionate**					
**Succinate**	P				1
**Hexose**					
**Lactate**					
**Formate**					
**Ethanol**					
**Butyrate**					
**other**					
**MFG4**					
**Pathway**	5				
	Type	K	y	μ_{max}	stoichiometry
**units**	none	g/L	g/g	/h	mol
**Protein**					
**NSP**	S	0.01	0.286	0.5	6
**RS**	S	0.01	0.333	0.5	6
**Sugars**	S	0.001	0.333	0.5	6
**H2**					

**CO2**					
**CH4**					
**H2O**	Sw				1
**Acetate**	P				10
**Propionate**					
**Succinate**					
**Hexose**					
**Lactate**	P				4
**Formate**	P				2
**Ethanol**	P				1
**Butyrate**					
**other**					

**MFG5**						
**Pathway**	6					
	Type	*K*	*y*	μ_{max}	stoichiometry	Fraction of fermentation
**units**	none	g/L	g/g	/h	mol	none
**Protein**						0.9
**NSP**	S	0.00045	0.55	0.55	2	
**RS**	S	0.00045	0.55	0.55	2	

**Sugars**	S	0.00045	0.55	0.55	2	
**H2**	P				2	
**CO2**	P				4	
**CH4**						
**H2O**	P				2	
**Acetate**	Sb	0.00045			2	
**Propionate**						
**Succinate**						
**Hexose**						
**Lactate**						
**Formate**						
**Ethanol**						
**Butyrate**	P				3	
**other**						
**MFG6**						
**Pathway**	7					
	Type	*K*	*y*	μ_{max}	stoichiometry	Fraction of fermentation
**units**	none	g/L	g/g	/h	mol	none

**Protein**						0.8
**NSP**	S	0.00045	0.55	0.4	6	
**RS**	S	0.00045	0.55	0.4	6	
**Sugars**	S	0.00045	0.55	0.4	6	
**H2**						
**CO2**	P				4	
**CH4**						
**H2O**	P				4	
**Acetate**	Sb	0.00045			4	
**Propionate**						
**Succinate**						
**Hexose**						
**Lactate**	P				2	
**Formate**	P				6	
**Ethanol**						
**Butyrate**	P				7	
**other**						

MFG7

**Pathway**	8					9				
	Type	*K*	*y*	μ_{max}	stoichiometry	Type	*K*	*y*	μ_{max}	stoichiometry
**units**	none	g/L	g/g	/h	mol	none	g/L	g/g	/h	mol
**Protein**										
**NSP**	S	0.0015	0.286	1	3					
**RS**	S	0.0015	0.333	1	3					
**Sugars**	S	0.0015	0.333	1	3					
**H2**										
**CO2**	P				2	P				1
**CH4**										
**H2O**	P				2	P				1
**Acetate**	P				2	P				1
**Propionate**	P				4	P				2
**Succinate**										
**Hexose**										
**Lactate**						Se	0.0015	0.111	1	3

**Formate**										
**Ethanol**										
**Butyrate**										
**other**										
**MFG8**										
**Pathway**	10					11				
	Type	*K*	*y*	μ_{max}	stoichiometry	Type	*K*	*y*	μ_{max}	stoichiometry
**units**	none	g/L	g/g	/h	mol	none	g/L	g/g	/h	mol
**Protein**										
**NSP**	S	0.00045	0.5	0.55	10					
**RS**	S	0.00045	0.5	0.55	10					
**Sugars**	S	0.00045	0.5	0.55	10					
**H2**	P				10	P				2
**CO2**	P				8	P				4
**CH4**										
**H2O**	Sw				2	P				2
**Acetate**	P				2	Se	0.00045			2

**Propionate**										
**Succinate**										
**Hexose**										
**Lactate**						Se	0.00045	0.111	0.55	4
**Formate**	P				12					
**Ethanol**										
**Butyrate**	P				9	P				3
**other**										

**MFG9**															
**Pathway**	12					13					14				
	Type	*K*	*y*	μ_{max}	stoichiometry	Type	*K*	*y*	μ_{max}	stoichiometry	Type	*K*	*y*	μ_{max}	stoichiometry
**units**	none	g/L	g/g	/h	mol	none	g/L	g/g	/h	mol	none	g/L	g/g	/h	mol
**Protein**															

**NSP**	S	0.005	0.286	0.008	1						S	0.005	0.286	0.008	1
**RS**	S	0.005	0.333	0.008	1						S	0.005	0.333	0.008	1
**Sugars**	S	0.005	0.333	0.027	1						S	0.005	0.333	0.027	1
**H2**						Se	0.005			4	P				2
**CO2**						Se	0.005	0.03	0.003	2	P				2
**CH4**															
**H2O**						P				2					

**Acetate**	P				3	P				1	P				3
**Propionate**															
**Succinate**															
**Hexose**															
**Lactate**															
**Formate**											Se	0.005			2
**Ethanol**															
**Butyrate**															
**other**															

**MFG10**										
**Pathway**	15					16				
	Type	*K*	*y*	μ_{max}	stoichiometry	Type	*K*	*y*	μ_{max}	stoichiometry
**units**	none	g/L	g/g	/h	mol	none	g/L	g/g	/h	mol

**Protein**										
**NSP**										
**RS**										
**Sugars**										
**H2**	Se	0.005			4					
**CO2**	Se	0.005	0.03	0.000305	1	P				3
**CH4**	P				1	P				1
**H2O**	P				2	P				2
**Acetate**										
**Propionate**										
**Succinate**										
**Hexose**										
**Lactate**										
**Formate**						Se	0.00045	0.00724	0.000305	4
**Ethanol**										
**Butyrate**										
**other**										

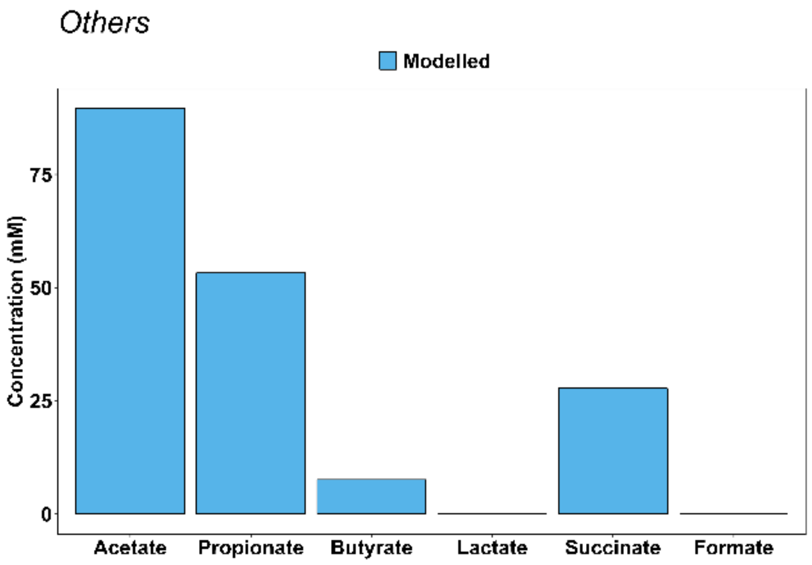

Figure 17. Predicted fermentation pattern of *Others* growing in batch for 50 hours. This data frame was modelled through 16 pathways based on the MFG description described above (1) and with modelling parameters established through reverse modelling via comparison to a bioreactor experiment, as described in the main manuscript. Starting modelled conditions: sugars 2g/l, resistant starch 4.32g/l, non-starch polysaccharides 7g/l, protein 15g/l, H_2O non limiting, pH 6.25, *Others* 0.1g/l.

19 References

1. Kettle H, Holtrop G, Louis P, Flint HJ. 2018. microPop: Modelling microbial populations and communities in R. Methods Ecol Evol 9:399–409.

2. Crespo-Piazuelo D, Estellé J, Revilla M, Criado-Mesas L, Ramayo-Caldas Y, Óvilo C, Fernández AI, Ballester M, Folch JM. 2018. Characterization of bacterial microbiota compositions along the intestinal tract in pigs and their interactions and functions. Sci Rep 8:1–12.

3. Yang H, Huang X, Fang S, He M, Zhao Y, Wu Z, Yang M, Zhang Z, Chen C, Huang L. 2017. Unraveling the fecal microbiota and metagenomic functional capacity associated with feed efficiency in pigs. Front Microbiol 8:1–11.

4. Kim JN, Méndez–García C, Geier RR, Iakiviak M, Chang J, Cann I, Mackie RI. 2017. Metabolic networks for nitrogen utilization in Prevotella ruminicola 23. Sci Rep 7:1–11.

5. Marounek M, Dušková D. 1999. Metabolism of pectin in rumen bacteria Butyrivibrio fibrisolvens and Prevotella ruminicola. Lett Appl Microbiol 29:429–433.

6. Kettle H, Louis P, Holtrop G, Duncan SH, Flint HJ. 2015. Modelling the emergent dynamics and major metabolites of the human colonic microbiota. Environ Microbiol 17:1615–1630.

7. Russell JB, Dombrowski DB. 1980. Effect of pH on the efficiency of growth by pure cultures of rumen bacteria in continuous culture. Appl Environ Microbiol 39:604–610.

8. Kovar L, Savka O, Duskova D, Marounek M. 1999. Fermentation of glucose and xylose in Prevotella ruminicola AR29. J Anim Feed Sci 8:115–122.

9. Marchandin H, Juvonen R, Haikara A. 2009. Megasphaera, p. 1082–1090. *In* de Vos, P, Garrity, GM, Jones, D, Krieg, NR, Ludwig, W, Rainey, FA, Schleifer, K-H, Whitman, WB (eds.), Bergey's Manual ® of Systematic Bacteriology Second Edition Volume Three The FirmicutesSecond Edi. Springer New York.

10. Kajihara Y, Yoshikawa S, Cho Y, Ito T, Miyamoto H, Kodama H. 2017. Preferential isolation of Megasphaera elsdenii from pig feces. Anaerobe 48:160–164.

11. Xiao-lin J, Yong S, Wei-yun Z. 2016. Fermentation characteristics of Megasphaera elsdenii J6 derived from pig feces on different lactate isomers. J Integr Agric 15:1575–1583.

12. Krieg NR, Staley JT, Brown DR, Hedlund BP, Paster BJ, Ward NL, Ludwig W, Whitman WB. 2015. Rikenellaceae fam. nov., p. 54–56. *In* Krieg, NR, Staley, JT, Brown, DR, Hedlund, BP, Paster, BJ, Ward, NL, Ludwig, W, Whitman, WB (eds.), Bergey's Manual of Systematics of Archaea and BacteriaSecond Edi. Springer New York.

13. Song Y, Liu C, Finegold SM. 2015. Bacteroides, p. 1–24. *In* Bergey's Manual of Systematics of Archaea and Bacteria. American Cancer Society.

14. Allison C, Macfarlane GT. 1989. Influence of pH, nutrient availability, and growth rate on amine production by Bacteroides fragilis and Clostridium perfringens. Appl Environ Microbiol 55:2894–2898.

15. Thanantong N, Edwards S, Sparagano OAE. 2006. Characterization of Lactic Acid Bacteria and Other Gut Bacteria in Pigs by a Macroarraying Method. Ann N Y Acad Sci 1081:276–279.

16. Devriese LA, Kilpper-Balz R, Schleifer2 AKH. 1988. Streptococcus hyointestinalis sp. nov. from the Gut of Swine. INTERNATIONAL JOURNAL OF SYSTEMATIC BACTERIOLOGY International Union of Microbiological Societies 38:440–441.

17. Whiley RA, Hardie JM. 2015. Streptococcus, p. 1–86. *In* Bergey's Manual of Systematics of Archaea and Bacteria. American Cancer Society.

18. Russell JB, Hino T. 1985. Regulation of Lactate Production in Streptococcus bovis: A Spiraling Effect That Contributes to Rumen Acidosis. J Dairy Sci 68:1712–1721.

19. Chen L, Luo Y, Wang H, Liu S, Shen Y, Wang M. 2016. Effects of glucose and starch on lactate production by newly isolated Streptococcus bovis S1 from Saanen goats. Appl Environ Microbiol 82:5982–5989.

20. Beal C, Louvet P, Corrieu G. 1989. Influence of controlled pH and temperature on the growth and acidification of pure cultures of Streptococcus thermophilus 404 and Lactobacillus bulgaricus 398. Appl Microbiol Biotechnol 32:148–154.

21. Roos S, Karner F, Axelsson L, Jonsson H. 2000. Lactobacillus mucosae sp. nov., a new species with in vitro mucus- binding activity isolated from pig intestine. Int J Syst Evol Microbiol 50:251–258.

22. Vogel RF, Bocker G, Stolz P, Ehrmann M, Fanta D, Ludwig W, Pot B, Kersters K, Schleifer KH, Hammes WP. 1994. Identification of lactobacilli from sourdough and description of Lactobacillus pontis sp. nov. Int J Syst Bacteriol 44:223–229.

23. Hou C, Zeng X, Yang F, Liu H, Qiao S. 2015. Study and use of the probiotic Lactobacillus reuteri in pigs: a review. J Anim Sci Biotechnol 6:14.

24. Burgé G, Saulou-Bérion C, Moussa M, Allais F, Athes V, Spinnler HE. 2015. Relationships between the use of Embden Meyerhof pathway (EMP) or Phosphoketolase pathway (PKP) and lactate production capabilities of diverse Lactobacillus reuteri strains. Journal of Microbiology 53:702–710.

25. de Vries W, Kapteijn WMC, van der Beek EG, Stouthamer AH. 1970. Molar growth yields and fermentation balances of Lactobacillus casei L3 in batch cultures and in continuous cultures. J Gen Microbiol 63:333–345.

26. El-Ziney MG, Arneborg N, Uyttendaele M, Debevere J, Jakobsen M. 1998. Characterization of growth and metabolite production of Lactobacillus reuteri during glucose/glycerol cofermentation in batch and continuous cultures. Biotechnol Lett 20:913–916.

27. Årsköld E, Lohmeier-Vogel E, Cao R, Roos S, Rådström P, van Niel EWJ. 2008. Phosphoketolase pathway dominates in Lactobacillus reuteri ATCC 55730 containing dual pathways for glycolysis. J Bacteriol 190:206–212.

28. Palmfeldt J, Hahn-Hägerdal B. 2000. Influence of culture pH on survival of Lactobacillus reuteri subjected to freeze-drying. Int J Food Microbiol 55:235–238.

29. Klantschitsch T, Spillmann H, Puhan Z. 1996. Lactobacillus Reuteri: A Newcomer in Dairy Technology. Mljekarstvo 46:183–196.

30. Downes J, Dewhirst FE, Tanner ACR, Wade WG. 2013. Description of Alloprevotella rava gen. nov., sp. nov., isolated from the human oral cavity, and reclassification of Prevotella tannerae Moore et al. 1994 as Alloprevotella tannerae gen. nov., comb. nov. Int J Syst Evol Microbiol 63:1214–1218.

31. Moore L V, Johnson JL, Moore WE. 1994. Descriptions of Prevotella tannerae sp. nov. and Prevotella enoeca sp. nov. from the human gingival crevice and emendation of the description of Prevotella zoogleoformans. Int J Syst Bacteriol 44:599–602.

32. Sato Y, Kuroki Y, Oka K, Takahashi M, Rao S, Sukegawa S, Fujimura T. 2019. Effects of dietary supplementation with Enterococcus faecium and Clostridium butyricum, either alone or in combination, on growth and fecal microbiota composition of post-weaning pigs at a commercial farm. Front Vet Sci 6.

33. Guo P, Zhang K, Ma X, He P. 2020. Clostridium species as probiotics: potentials and challenges. J Anim Sci Biotechnol 11:1–10.

34. Rainey FA, Hollen BJ, Small A. 2009. Clostridium, p. 738–828. *In* de Vos, P, Garrity, GM, Jones, D, Krieg, NR, Ludwig, W, Rainey, FA, Schleifer, K-H, Whitman, WB (eds.), Bergey's Manual of Systematic Bacteriology Second Edition Volume Three The FirmicutesSecond Edi. Springer New York.

35. Schink B, Ward JC, Zeikusl JG. 1981. Microbiology of wetwood: importance of pectin degradation and clostridium species in living trees. Appl Environ Microbiol 42:526–532.

36. Detman A, Mielecki D, Chojnacka A, Salamon A, Błaszczyk MK, Sikora A. 2019. Cell factories converting lactate and acetate to butyrate: Clostridium butyricum and microbial communities from dark fermentation bioreactors. Microb Cell Fact 18:1–12.

37. He G, Kong Q, Chen Q, Ruan H. 2005. Batch and fed-batch production of butyric acid by clostridium butyricum ZJUCB. J Zhejiang Univ Sci B 6:1076–1080.

38. Nordhoff M, Taras D, Macha M, Tedin K, Busse HJ, Wieler LH. 2005. Treponema berlinense sp. nov. and Treponema porcinum sp. nov., novel spirochaetes isolated from porcine faeces. Int J Syst Evol Microbiol 55:1675–1680.

39. Cwyk WM, Canale-Parola E. 1979. Treponema succinifaciens sp. nov., an anaerobic spirochete from the swine intestine. Arch Microbiol 122:231–239.

40. Louis P, Flint HJ. 2009. Diversity, metabolism and microbial ecology of butyrate-producing bacteria from the human large intestine. FEMS Microbiol Lett 294:1–8.

41. Duncan SH, Hold GL, Harmsen HJM, Stewart CS, Flint HJ. 2002. Growth requirements and fermentation products of Fusobacterium prausnitzii, and a proposal to reclassify it as Faecalibacterium prausnitzii gen. nov., comb. nov. Int J Syst Evol Microbiol 52:2141–2146.

42. Duncan SH, Barcenilla A, Stewart CS, Pryde SE, Flint HJ. 2002. Acetate utilization and butyryl coenzyme A (CoA): Acetate-CoA transferase in butyrate-producing bacteria from the human large intestine. Appl Environ Microbiol 68:5186–5190.

43. Lopez-Siles M, Duncan SH, Garcia-Gil LJ, Martinez-Medina M. 2017. Faecalibacterium prausnitzii: From microbiology to diagnostics and prognostics. ISME Journal https://doi.org/10.1038/ismej.2016.176.

44. Foditsch C, Santos TMA, Teixeira AGV, Pereira RVV, Dias JM, Gaeta N, Bicalho RC. 2014. Isolation and characterization of faecalibacterium prausnitzii from calves and piglets. PLoS One 9.

45. Bryant MP, Small N. 1955. Characteristics of two new genera of anaerobic curved rods isolated from the rumen of cattle. J Bacteriol 22–26.

46. O’Herrin SM, Kenealy WR. 1993. Glucose and carbon dioxide metabolism by Succinivibrio dextrinosolvens. Appl Environ Microbiol 59:748–755.

47. Bryant MP. 2007. Succinivibrio, p. 581–582. *In* Staley, JT, Boone, DR, Brenner, DJ, de Vos, P, Garrity, GM, Goodfellow, M, Krieg, NR, Rainey, FA, Schleifer, K-H (eds.), Bergey’s Manual of Systematic Bacteriology, Second Edition, Volume 2: The Proteobacteria, part B: The GammaproteobacteriaSecond Edi. Springer New York.

48. Hailemariam S, Zhao S, Wang J. 2020. Complete Genome Sequencing and Transcriptome Analysis of Nitrogen Metabolism of Succinivibrio dextrinosolvens Strain Z6 Isolated From Dairy Cow Rumen. Front Microbiol 11.

49. Kamlage B, Gruhl B, Blaut M. 1997. Isolation and characterization of two new homoacetogenic hydrogen-utilizing bacteria from the human intestinal tract that are closely related to Clostridium coccoides. Appl Environ Microbiol 63:1732–1738.

50. Park S-K, Kim M-S, Bae J-W. 2013. Blautia faecis sp. nov., isolated from human faeces. Int J Syst Evol Microbiol 65:599–603.

51. Durand GA, Pham T, Ndongo S, Traore SI, Dubourg G, Lagier JC, Michelle C, Armstrong N, Fournier PE, Raoult D, Million M. 2017. Blautia massiliensis sp. nov., isolated from a fresh human fecal sample and emended description of the genus Blautia. Anaerobe 43:47–55.

52. D'hoe K, Vet S, Faust K, Moens F, Falony G, Gonze D, Lloréns-Rico V, Gelens L, Danckaert J, de Vuyst L, Raes J. 2018. Integrated culturing, modeling and transcriptomics uncovers complex interactions and emergent behavior in a three-species synthetic gut community. Elife 7:e37090.

53. Louis P, Hold GL, Flint HJ. 2014. The gut microbiota, bacterial metabolites and colorectal cancer. Nat Rev Microbiol 12:661–672.

54. Müller V. 2003. Energy Conservation in Acetogenic Bacteria. Appl Environ Microbiol 69:6345–6353.

55. Drake HL, Gößner AS, Daniel SL. 2008. Old acetogens, new light. Ann N Y Acad Sci 1125:100–128.

56. Bernalier A, Willems A, Leclerc M, Rochet V, Collins MD. 1996. Ruminococcus hydrogenotrophicus sp. nov., a new H2/CO2-utilizing acetogenic bacterium isolated from human feces. Arch Microbiol 166:176–183.

57. Liu C, Finegold SM, Song Y, Lawson PA. 2008. Reclassification of Clostridium coccoides, Ruminococcus hansenii, Ruminococcus hydrogenotrophicus, Ruminococcus luti, Ruminococcus productus and Ruminococcus schinkii as Blautia coccoides gen. nov., comb. nov., Blautia hansenii comb. nov., Blautia hydroge. Int J Syst Evol Microbiol 58:1896–1902.

58. D'hoe K, Vet S, Faust K, Moens F, Falony G, Gonze D, Lloréns-Rico V, Gelens L, Danckaert J, de Vuyst L, Raes J. 2018. Integrated culturing, modeling and transcriptomics uncovers complex interactions and emergent behavior in a three-species synthetic gut community. Elife 7.

59. Del Dot T, Osawa R, Stackebrandt E. 1993. Phascolarctobacterium faecium gen. nov, spec. nov., a Novel Taxon of the Sporomusa Group of Bacteria. Syst Appl Microbiol 16:380–384.

60. Watanabe Y, Nagai F, Morotomi M. 2011. Characterization of Phascolarctobacterium succinatutens sp. Nov., an asaccharolytic, succinate-utilizing bacterium isolated from human feces. Appl Environ Microbiol 78:511–518.

61. Shigeno Y, Kitahara M, Shime M, Benno Y. 2019. Phascolarctobacterium wakonense sp. nov., isolated from common marmoset (Callithrix jacchus) faeces. Int J Syst Evol Microbiol 69:1941–1946.

62. Ezaki T. 2009. Ruminococcus, p. 1016–1018. *In* De Vos, P, Garrity, GM, Jones, D, Krieg, NR, Ludwig, W, Rainey, FA, Schleifer, K-H, Whitman, WB (eds.), Bergey's Manual ® of Systematic Bacteriology Second Edition Volume Three The FirmicutesSecond Edi. Springer New York.

63. Laverde Gomez JA, Mukhopadhya I, Duncan SH, Louis P, Shaw S, Collie-Duguid E, Crost E, Juge N, Flint HJ. 2019. Formate cross-feeding and cooperative metabolic interactions revealed

by transcriptomics in co-cultures of acetogenic and amylolytic human colonic bacteria. Environ Microbiol 21:259–271.

64. Robert C, Bernalier-Donadille A. 2003. The cellulolytic microflora of the human colon: Evidence of microcrystalline cellulose-degrading bacteria in methane-excreting subjects. FEMS Microbiol Ecol 46:81–89.

65. Kettle H, Holtrop G, Louis P, Flint HJ. 2018. microPop: Modelling microbial populations and communities in R. Methods Ecol Evol 399–409.

66. Tan Z, Dong W, Ding Y, Ding X, Zhang Q, Jiang L. 2019. Changes in cecal microbiota community of suckling piglets infected with porcine epidemic diarrhea virus. PLoS One 14:1–14.

67. Sakamoto M, Benno Y. 2006. Reclassification of Bacteroides distasonis, Bacteroides goldsteinii and Bacteroides merdae as Parabacteroides distasonis gen. nov., comb. nov., Parabacteroides goldsteinii comb. nov and Parabacteroides merdae comb. nov. Int J Syst Evol Microbiol 56:1599–1605.

68. Wang YJ, Xu XJ, Zhou N, Sun Y, Liu C, Liu SJ, You X. 2019. Parabacteroides acidifaciens sp. Nov., isolated from human faeces. Int J Syst Evol Microbiol 69:761–766.

69. Yimagou EK, Dione N, Ngom II, Tall ML, Baudoin JP, Raoult D, Khalil JYB. 2020. Parabacteroides bouchesdurhonensis sp. nov., a new bacterium isolated from the stool of a healthy adult. New Microbes New Infect 34:100639.

70. Tan HQ, Li TT, Zhu C, Zhang XQ, Wu M, Zhu XF. 2012. Parabacteroides chartae sp. nov., an obligately anaerobic species from wastewater of a paper mill. Int J Syst Evol Microbiol 62:2613–2617.

71. Kitahara M, Sakamoto M, Tsuchida S, Kawasumi K, Amao H, Benno Y, Ohkuma M. 2013. Parabacteroides chinchillae sp. nov., isolated from chinchilla (Chincilla lanigera) faeces. Int J Syst Evol Microbiol 63:3470–3474.

72. Kim H, Im WT, Kim M, Kim D, Seo YH, Yong D, Jeong SH, Lee K. 2018. Parabacteroides chongii sp. nov., isolated from blood of a patient with peritonitis. Journal of Microbiology 56:722–726.

73. Sakamoto M, Tanaka Y, Benno Y, Ohkuma M. 2015. Parabacteroides faecis sp. nov., isolated from human faeces. Int J Syst Evol Microbiol 65:1342–1346.

74. Song Y, Liu C, Lee J, Bolanos M, Vaisanen ML, Finegold SM. 2005. “ Bacteroides goldsteinii sp. nov.” Isolated from Clinical Specimens of Human Intestinal Origin. J Clin Microbiol 43:4522–4527.

75. Sakamoto M, Suzuki N, Matsunaga N, Koshihara K, Seki M, Komiya H, Benno Y. 2009. Parabacteroides gordonii sp. nov., isolated from human blood cultures. Int J Syst Evol Microbiol 59:2843–2847.

76. Sakamoto M, Kitahara M, Benno Y. 2007. Parabacteroides johnsonii sp. nov., isolated from human faeces. Int J Syst Evol Microbiol 57:293–296.

77. Bellali S, Lo CI, Naud S, Fonkou MDM, Armstrong N, Raoult D, Fournier PE, Fenollar F. 2019. Parabacteroides massiliensis sp. nov., a new bacterium isolated from a fresh human stool specimen. New Microbes New Infect 32:1–6.

78. Johnson JL, Moore WEC, Moore LVH. 1986. Bacteroides caccae sp. nov., Bacteroides merdae sp. nov., and Bacteroides stercoris sp. nov. Isolated from human feces. Int J Syst Bacteriol 36:499–501.

79. Benabdelkader S, Naud S, Lo CI, Fadlane A, Traore SI, Aboudharam G, La Scola B. 2020. Parabacteroides pacaensis sp. nov. and Parabacteroides provencensis sp. nov., two new species identified from human gut microbiota. New Microbes New Infect 34:1–6.

80. Bilen M, Cadoret F, Daoud Z, Fournier PE, Raoult D. 2016. Parabacteroides timonensis sp. nov., identified in human stool. Hum Microb J 2:1–2.

81. Moodley A, Guardabassi L. 2009. Transmission of IncN plasmids carrying blaCTX-M-1 between commensal escherichia coli in pigs and farm workers. Antimicrob Agents Chemother 53:1709–1711.

82. Rasko DA, Rosovitz MJ, Myers GSA, Mongodin EF, Fricke WF, Gajer P, Crabtree J, Sebaihia M, Thomson NR, Chaudhuri R, Henderson IR, Sperandio V, Ravel J. 2008. The pangenome structure of Escherichia coli: Comparative genomic analysis of E. coli commensal and pathogenic isolates. J Bacteriol 190:6881–6893.

83. Heo JM, Opapeju FO, Pluske JR, Kim JC, Hampson DJ, Nyachoti CM. 2013. Gastrointestinal health and function in weaned pigs: A review of feeding strategies to control post-weaning diarrhoea without using in-feed antimicrobial compounds. J Anim Physiol Anim Nutr (Berl) 97:207–237.

84. Alam KY, Clark DP. 1989. Anaerobic Fermentation Balance of Escherichia coli as Observed by In Vivo Nuclear Magnetic Resonance Spectroscopy. J Bacteriol 171:6213–6217.

85. Clark DP. 1989. The fermentation pathways of *Escherichia coli*. FEMS Microbiol Lett 63:223–234.

86. Nutting LA, Carson SF. 1952. Lactic acid fermentation of xylose by Escherichia coli. J Bacteriol 53:575–580.

87. Sauer U, Lasko DR, Fiaux J, Hochuli M, Glaser R, Szyperski T, Wüthrich K, Bailey JE. 1999. Metabolic flux ratio analysis of genetic and environmental modulations of Escherichia coli central carbon metabolism. J Bacteriol 181:6679–88.

88. Adler J, Templeton B. 1967. The effect of environmental conditions on the motility of Escherichia coli. J Gen Microbiol 46:175–184.

89. Bosshard PP, Bosshard, P. P. 2002. Turicibacter, p. 1314. *In* Bergey's Manual of Systematics of Archaea and Bacteria. John Wiley & Sons, Ltd, Chichester, UK.

90. Bosshard PP, Zbinden R, Altwegg M. 2002. Turicibacter sanguinis gen. nov., sp. nov., a novel anaerobic, Gram-positive bacterium. Int J Syst Evol Microbiol 52:1263–1266.
