# Supplementary material for: Estimating the contribution of the porcine fecal core microbiota to metabolite production via mathematical modeling and in vitro fermentation
Source: mSystems. 2023 Dec 7;9(1):e00366-23. doi: 10.1128/msystems.00366-23 (PMC10805034; doi:10.1128/msystems.00366-23)
Supplement: Tables S21 and S22 — Composition of the medium used during the continuous fermentation experiment. [file msystems.00366-23-s0002.pdf]

Medium composition used through the continuous fermentation experiment.

S 21. Medium composition (1).

**Component**	**Concentration (g/l)**
Corn starch	4.32
Pectin (citrus)	2
Xylan (corn core)	2
Arabinogalactan (beechwood)	2
Guar gum	2
Soy peptone	13
Yeast extract	4.50
Mucin (from porcine stomach)	4.00
L-cysteine HCL monohydrate	0.8
Vitamin solution	1 ml
Resazurin (0.01%)	1 ml

**Component**	**Concentration (g/l)**
Bile extract porcine	0.4
KH_2PO_4	0.5
$NaHCO_3$	1.5
NaCl	4.5
KCl	4.5
$MgSO_4$	0.64
$CaCl_2 \cdot 2H_2O$	0.15
$MnCl_2 \cdot 4H_2O$	0.20
Hemin solution (0.05g/ml)	0.05
Tween 80	1.00

S 22. Vitamin solution used in the complex medium (2).

Component	Concentration (mg/l)
Menadione	1.0
Biotin	2.0
Pantothenate	10.0
Nicotinamide	5.0
Vitamin B_{12}	0.5
Thiamine	4.0
P-Aminobenzoic acid	5.0

References

1. Tanner SA, Berner AZ, Rigozzi E, Grattepanche F, Chassard C, Lacroix C. 2014. In vitro continuous fermentation model (PolyFermS) of the swine proximal colon for simultaneous testing on the same gut microbiota. PLoS One 9:e94123.

2. Kim B-S, Kim JN, Cerniglia CE. 2011. In vitro culture conditions for maintaining a complex population of human gastrointestinal tract microbiota. Journal of biomedicine & biotechnology 2011:838040.
